# Supplementary material for: Feasibility of a pulmonary rehabilitation programme for patients with symptomatic chronic obstructive pulmonary disease in Georgia: a single-site, randomised controlled trial from the Breathe Well Group
Source: BMJ Open. 2022 Sep 23;12(9):e056902. doi: 10.1136/bmjopen-2021-056902 (PMC9511586; doi:10.1136/bmjopen-2021-056902)
Supplement: Supplementary data [file bmjopen-2021-056902supp001.pdf]

Supplementary tables and figures

Table S1: List of education topics delivered as part of the PR programme

COPD as a global problem
What you and your family should know about COPD
Breathing and breath physiology
Tobacco, its impact on health and smoking cessation
Benefits of physical activity
Regular exercises
Clearance of the respiratory system: keeping clean lungs
Energy conservation: additional energy
Home oxygen therapy
COPD and swallowing
Management of depression, anxiety and fears during COPD
Nutrition recommendations for COPD patients
Self-assessment and self-management
Loving relationships/Sexuality
COPD and incontinence
COPD and treatment, drugs

Components of exercise rehabilitation

Centre-based supervised exercise sessions twice weekly for 8-weeks

Advice to perform an unsupervised exercise session each week

Exercise tailored to the individual, using the modified Borg score to inform exercise prescription

Endurance and strength training

Training of upper and lower limbs

Table S2: Topics covered in training the rehabilitation specialist

Overview of pulmonary rehabilitation
information about the incremental shuttle walk test (ISWT) test and practical
Patient assessment
Difference between cardiac and pulmonary rehabilitation.
Format of a pulmonary rehab programme.
Exercise prescription.
Practical session of the exercises.
Education sessions:<br>anxiety and breathlessness,<br>airway clearance techniques (ACBT),<br>relaxation
Discharge from rehab to include:<br>Assessment,<br>Goal setting<br>Maintenance exercise.

Table S3: Topic guides for qualitative interviews

Patient interview guide

<u>Feasibility trial of an adapted pulmonary rehabilitation programme in Georgia</u>

1) I'd like you to tell me about your experience of taking part in this study.

2) Can you tell be about how you found out about the study?

   Prompt:

   a. What were your thoughts when you were invited to participate? Why did you decide to participate?

   b. What ideas did you have about how it would affect your condition?

   c. Did you think you would gain anything?

   d. Did you have any concerns about participating?

3) What were your expectations of the PR programme prior to the start?

   Prompts:
   a. Had you ever heard of it before?
   b. What did you think would happen in the programme?
   c. How did you feel about exercise before you started the programme?

      i. What type of exercise did you feel able to do then? Why?

      ii. And what did you feel that you couldn't do? Why?

   d. What did you expect to get from the programme? Did you think anything would change?

4) What was your experience of the research assessment visits?

   Prompt:

   a. What did you think of the questionnaire content/length?

   b. What did you think of the shuttle walk test?

      i. Did you understand what to do?
      ii. How easy was it to do?

5) Can you tell me about the pre-rehab session?

Prompt:

a. What role did it play with regard to your understanding of the PR program?

b. What was positive about it?

c. What was less helpful?

6) Can you tell me about your experience of the rehabilitation?

Prompt:

a. What was good about it?
b. What was not so good?

7) How many of the sessions were you able to attend?

Prompt:

a. If not all of them, why not?
(Location, transport, access, cost, season, illness, family matters, holidays)

Barriers to attendance:

b. If did attend, what difficulties did you face in attending?
c. Why did you still try to keep on going?

8) What did you think of the education sessions?

Prompt:

a. COPD disease, causes [looking for smoking], treatment, exacerbations, exercise, self-management, diet, lifestyle related information [anything else]?
b. Was there anything about the education sessions/ that you received which you felt was not relevant for your condition and situation?

9) In what ways did the program influence your everyday lifestyle?

a. Have you done any of the exercises at home? If yes, are you still continuing to do them?
b. Are you a smoker? [if not already stated]
    i. Did you learn anything about smoking and COPD? [if yes]
    ii. What did you learn?

10) Did you incur any costs in attending the rehabilitation?

a. Have you made any purchases as a result of doing the rehabilitation?

Prompt: e.g. equipment? Shoes? Clothing?

11) What changes would you make to improve PR?

Prompt:

a. Time, location, content.
b. Was there anything you would like to add to the sessions (physical exercises of some kind, information)?
c. Anything which you think should not have been included?

12) How widely do you think pulmonary rehab should be available? *If yes…*

Prompt

a. How do you think it should be paid for? [is this something you would pay for]
b. Where and when should the sessions be?

13) What do you think are the advantages of PR in comparison with other ways of getting exercise? And the disadvantages?

14) So what are you going to do now that the rehabilitation programme is over?

Prompts around continued exercise

a. If not already discussed: Have you thought about exercising at home?
b. Do you think you will do anything to change your physical activity level and approaches now that the PR classes are over?

Ending the interview: Is there anything else you would like to say? Any important issues not raised? Thank participant for their time.

Specialists' Interview topic guide - Georgia

Feasibility trial of an adapted pulmonary rehabilitation programme in Georgia

1) Before participating in this study, what were your thoughts on pulmonary rehab?
   Prompts:
   a. What did you know about PR? How detailed was the information you possessed before on PR? What impact you thought it would have on patients?
   b. Impact on health? Other?
2) What are your views about pulmonary rehab (open question)
   Prompts:
   a. What experience did you have of PR?
   b. Two consecutive questions:
      a. Can you describe the positive aspects of PR from your point of view?
      b. Can you describe negative aspects of PR from your point of view?
      c. From your point of view what kind of impact does pulmonary rehab have on health?

3) Why did you agree to support this pulmonary rehab research study?
   Prompt:
   a. What were your thoughts when you were invited to take part?

4) How did you find the experience of providing PR?
   Prompt:
   a) In your opinion, as a physiotherapist what did you get from your experience?
   b) What was good about it?
   c) What was not so good?

5) How did patients seem to receive this programme?
   a) What was good?
   b) What was not so good?

6) How would you describe the effects of the PR for the patients?
   a. What benefits did you see?
   b. What challenges did you observe?

7) Can you tell me about the practical side of delivering the programme?
   a. What worked well?
   b. What didn't work so well?
   c. And from the research point of view? (was it a difficult, interesting – the meetings, the paperwork, fitting the PR appointments in around your everyday work schedule etc?)

8) What did you enjoy about delivering the programme?

9) Are there any changes you think should be made to the programme?

    Prompt:

    a. Time, location, content.
    b. Was there anything you would like to add to the sessions?
    c. Anything you would like to take out?

Future:

10) Do you think pulmonary rehab should be widely available?

    If yes…

    a) Where and when should the sessions be?
    b) How do you think it should be paid for?

    If no….

    a) Can you name the reasons why?

11) How would you feel if you were asked to run regular PR programs?

12) How interesting do you think this topic is for physiotherapists?

    Prompt:

    a) For what reasons?

13) Can you tell me about any conversations you had about PR with your colleague physiotherapists?

    Prompt:

    a) How would you describe their attitude towards PR?
    b) If you have not had any conversation, why do you think this topic never came up in the discussion?

14) From your point of view how will your participation in the study affect your practice?

Ending the interview: Is there anything else you would like to say? Any important issues not raised? Thank participant for their time.

Table S4: Costs of Pulmonary rehabilitation (PR) delivery

Type of cost	Units	Unit cost (GBP)	Total cost (GBP)
**Equipment costs:**			
TheraBand Resistive	5	£4.60	£23.00
Theraband Resistive	5	£5.00	£25.00
Theraband Resistive	5	£4.40	£4.00
Stepbox 2x	2	£15.00	£31.00
Weights 10kg	2	£13.00	£26.00
Weights 4kg	2	£5.30	£10.60
Medicine ball	2	£22.00	£44.00
Medicine ball	2	£14.00	£28.00
Water Dispenser	1	£96.00	£96.00
Stopwatch timer	8	£5.00	£40.00
Bottled water	3	£3.00	£9.60
Plastic cups	3	£0.40	£1.00
**Equipment total**		**£187.70**	**£338.20**
**Intervention development:**			
Translation patient video into Georgian	1		£212.00
Nutritionists' and psychologist's videos	1		£595.00
**Intervention development total**			**£807.00**
**Training:**			
Translation during PR training	1	£196.00	£196.00
Trainers - 4 days from UK	£7,200		£7,200.00
Attendees - 4 days	2	£320.00	£640.00
**Training total**			**£8,036.00**
**PR delivery**			41 sessions
Rehab specialist (£10/hr)	2 x 1.5 hours/session		
	30 mins x 7 per 16 week cycle	£10.00	£35.00
Senior respiratory specialist (£70/hr)	30 mins x 8 per 16 week cycle	£35.00	£140.00
Doctor (£40/hr)	30 mins x 1 per 16 week cycle	£20.00	£10.00
Baseline ISWT x 2	40 mins	£6.67	£200.10
Pre-rehab session	1 hr with rehab specialist	£10.00	£25.00
Exercise sessions	2 x 1.5 hours/session	£30.00	£1,230
Education	30 mins/session	£10.67	£437.47
Booklet	30	£1.80	£54.00
**Total PR delivery**			**£1,946.57**
**Total PR delivery per attendee**			**£81.11**

Table S5: Post-hoc per-protocol analysis: 8 week follow-up of intervention patients who attended 50% or more pulmonary rehabilitation sessions compared to usual care.

	Intervention n=9		Usual care n=26	
	**Mean (sd)**	**Mean change (sd) [95% CI]**	**Mean (sd)**	**Mean change (sd) [95% CI]**
SGRQ – Total[a]	27.23 (24.11)	-31.92 (23.57) [-50.05, -13.81]	48.04 (20.75)	-0.53 (17.96) [-8.12, 7.04]
SGRQ - Impacts	17.65 (20.91)	-30.03 (22.10) [-47.02, -13.03]	37.58 (23.17)	0.26 (22.85) [-9.17, 9.69]
SGRQ - Activity	37.79 (31.52)	-37.42 (26.43) [-57.74, -17.10]	58.60 (20.32)	1.13 (17.73) [-6.18, 8.45]
SGRQ - Symptoms	36.87 (29.66)	-27.94 (33.56) [-53.74, -2.15]	60.47 (25.68)	-4.16 (21.39) (-13.19, 4.86]
MRC dyspnoea score	2.22 (1.09)	-1.33 (1.00)	2.96 (1.09)	-0.24 (0.59)
CAT[b]	11.44 (6.44)	-9.11 (5.66)	18.32 (7.03)	-1.36 (5.00)
PHQ9 depression score[c]	2.11 (1.05)	-2.44 (3.20)	5.54 (5.09)	-0.29 (3.89)
GAD anxiety score[c]	1.66 (1.41)	-1.1 (3.51)	3.32 (3.47)	-0.28 (2.80)
Stanford Self Efficacy Score	8.16 (0.32)	2.07 (1.06)	6.9 (1.52)	0.24 (1.76)
ISWT[d]	337.77 (106.86)	146.66 (81.70)	247.6 (113.51)	-13.2 (110.66)
Self-reported number of exacerbations in the last 6 months	3.66 (1.00)	0.66 (0.86)	3.56 (0.96)	1.32 (1.67)
Total Physical Activity Scores. MET-minutes/week	1032.96 (1393.17)	27.39 (1718.72)	1634.68 (1532.01)	-1393.05 (2838.34)

[a]MCID 4 points; [b]CAT: -2 points; [c]MCID 20% reduction; [d]MCID 35-36m

Figure S1: Proportion of intervention group who attended pulmonary rehabilitation sessions

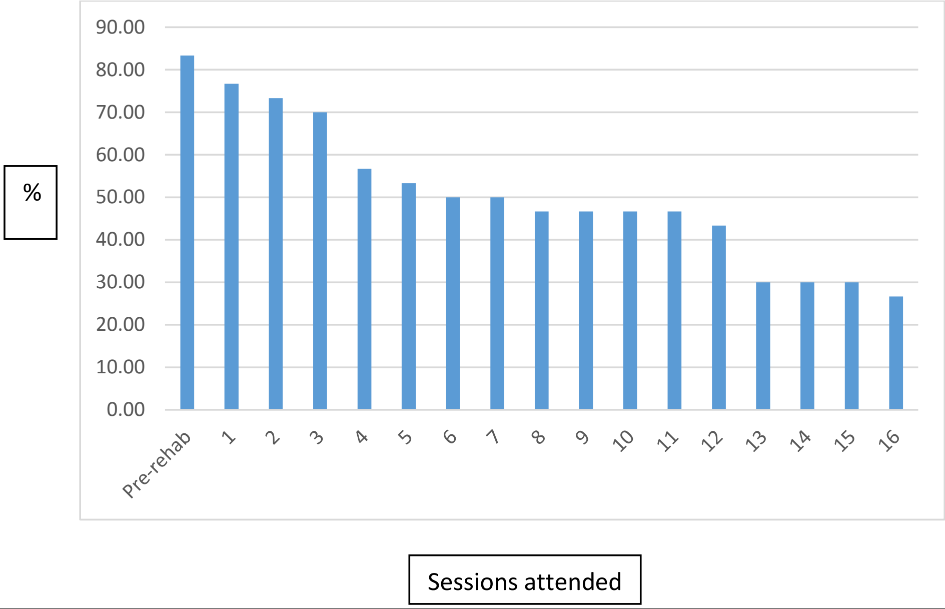

46.7% of participants attended at least 50% of planned sessions; 43% attended at least 75% of planned sessions.
