# Supplementary material for: Feasibility of a pulmonary rehabilitation programme for patients with symptomatic chronic obstructive pulmonary disease in Georgia: a single-site, randomised controlled trial from the Breathe Well Group
Source: BMJ Open. 2022 Sep 23;12(9):e056902. doi: 10.1136/bmjopen-2021-056902 (PMC9511586; doi:10.1136/bmjopen-2021-056902)
Supplement: Author's manuscript [file bmjopen-2021-056902.draft_revisions.pdf]

BMJ Open

BMJ Open is committed to open peer review. As part of this commitment we make the peer review history of every article we publish publicly available.

When an article is published we post the peer reviewers' comments and the authors' responses online. We also post the versions of the paper that were used during peer review. These are the versions that the peer review comments apply to.

The versions of the paper that follow are the versions that were submitted during the peer review process. They are not the versions of record or the final published versions. They should not be cited or distributed as the published version of this manuscript.

BMJ Open is an open access journal and the full, final, typeset and author-corrected version of record of the manuscript is available on our site with no access controls, subscription charges or pay-per-view fees (http://bmjopen.bmj.com).

If you have any questions on BMJ Open's open peer review process please email info.bmjopen@bmj.com

BMJ Open

Feasibility of a pulmonary rehabilitation (PR) programme for symptomatic COPD Patients in Georgia: a randomized controlled trial (RCT) from the Breathe Well Group

Journal:	*BMJ Open*
Manuscript ID	bmjopen-2021-056902
Article Type:	Original research
Date Submitted by the Author:	01-Sep-2021
Complete List of Authors:	Maglakelidze, Mariam; Georgian Respiratory Association; Petre Shotadze Tbilisi Medical Academy Ltd<br>Kurua, Ia<br>Maglakelidze, Nino<br>Maglakelidze, Tamaz; Ivane Javakhishvili Tbilisi State University Faculty of Medicine; Tbilisi State Medical University<br>Chkhaidze, Ivane; Georgian Respiratory Association; Tbilisi State Medical University Faculty of Medicine<br>Gogvadze, Ketevan<br>Chkhaidze, Natia; M. Iashvili Children's Central Hospital<br>Beadle, Helen; University Hospitals Birmingham NHS Foundation Trust, Department of Physiotherapy<br>Redden-Rowley, Kelly; Sandwell and West Birmingham Hospitals NHS Trust, iCares Directorate<br>Adab, Peymane; University of Birmingham College of Medical and Dental Sciences, Institute of Applied Health Research<br>Adams, Rachel; University of Birmingham College of Medical and Dental Sciences, Institute of Applied Health Research<br>Chi, Chunhua; Peking University First Hospital, General Practice Department, Peking University First Hospital, Beijing, 100034, China<br>Cheng, Kar Keung; University of Birmingham College of Medical and Dental Sciences, Institute of Applied Health Research<br>Cooper, Brendan; University Hospitals Birmingham NHS Foundation Trust, Lung Function & Sleep<br>Correia-de-Sousa, Jaime; University of Minho, Life and Health Sciences Research Institute; Horizonte Family Health Unit,<br>Dickens, Andrew; University of Birmingham, Institute of Applied Health Research<br>Enocson, Alexandra; University of Birmingham, Applied Health Research<br>Farley, Amanda; University of Birmingham College of Medical and Dental Sciences, Institute of Applied Health Research<br>Gale, Nicola K.; University of Birmingham College of Arts and Law, Health Services Management Centre<br>Jowett, Sue; University of Birmingham, Health Economics Unit<br>Martins, Sonia; ABC Medical School, Family Medicine<br>Rai, Kiran; University of Birmingham College of Medical and Dental Sciences, Institute of Applied Health Research<br>Sitch, Alice; University of Birmingham College of Medical and Dental Sciences, Institute of Applied Health Research; University Hospitals

	Birmingham NHS Foundation Trust and University of Birmingham, NIHR Birmingham Biomedical Research Centre<br>Stavrikj, Katarina; Medical Faculty, Centre for Family Medicine<br>Stelmach, Rafael; University of São Paulo<br>Turner, Alice; University of Birmingham College of Medical and Dental Sciences, Institute of Applied Health Research<br>Williams, Sian; National Heart and Lung Institute<br>Jordan, Rachel; University of Birmingham College of Medical and Dental Sciences, Institute of Applied Health Research<br>Jolly, Kate; University of Birmingham College of Medical and Dental Sciences, Institute of Applied Health Research

Keywords:	Thoracic medicine < INTERNAL MEDICINE, INTERNAL MEDICINE, GENERAL MEDICINE (see Internal Medicine)

SCHOLARONE™
Manuscripts

I, the Submitting Author has the right to grant and does grant on behalf of all authors of the Work (as defined in the below author licence), an exclusive licence and/or a non-exclusive licence for contributions from authors who are: i) UK Crown employees; ii) where BMJ has agreed a CC-BY licence shall apply, and/or iii) in accordance with the terms applicable for US Federal Government officers or employees acting as part of their official duties; on a worldwide, perpetual, irrevocable, royalty-free basis to BMJ Publishing Group Ltd ("BMJ") its licensees and where the relevant Journal is co-owned by BMJ to the co-owners of the Journal, to publish the Work in this journal and any other BMJ products and to exploit all rights, as set out in our licence.

The Submitting Author accepts and understands that any supply made under these terms is made by BMJ to the Submitting Author unless you are acting as an employee on behalf of your employer or a postgraduate student of an affiliated institution which is paying any applicable article publishing charge ("APC") for Open Access articles. Where the Submitting Author wishes to make the Work available on an Open Access basis (and intends to pay the relevant APC), the terms of reuse of such Open Access shall be governed by a Creative Commons licence – details of these licences and which Creative Commons licence will apply to this Work are set out in our licence referred to above.

Other than as permitted in any relevant BMJ Author's Self Archiving Policies, I confirm this Work has not been accepted for publication elsewhere, is not being considered for publication elsewhere and does not duplicate material already published. I confirm all authors consent to publication of this Work and authorise the granting of this licence.

For peer review only

Feasibility of a pulmonary rehabilitation (PR) programme for symptomatic COPD Patients in Georgia: a randomized controlled trial (RCT) from the Breathe Well Group

Maglakelidze Mariam[1,2], Ia Kurua[1], Nino Maglakelidze[1], Tamaz Maglakelidze[1,3*], Ivane Chkhaidze[1,4], Ketevan Gogvadze[1], Natia Chkhaidze[5], Helen Beadle[6], Kelly Redden-Rowley[7], Peymané Adab[8], Rachel Adams[8], Chunhua Chi[9], Kar Keung Cheng[8], Brendan G Cooper[10], Jaime Correia-de-Sousa[11,12], Andrew P Dickens[8], Alexandra Enocson[8], Amanda Farley[8], Nicola Gale[13], Sue Jowett[8], Sonia M Martins[14], Kiran Rai[8], Alice Sitch[8,15], Katarina Stavrikj[16], Rafael Stelmach[17], Alice Turner[8], Siân Williams[11], Rachel E Jordan[8], Kate Jolly[8]

*Corresponding author: Tamaz Maglakelidze (tmaglak@gmail.com);

[1]Georgian Respiratory Association, 2/6, Liubliana str, Tbilisi, Georgia.

[2]Petre Shotadze Tbilisi Medical Academy, 51/2 Ketevan Dedofali Ave, Tbilisi, 0144, Georgia.

[3]Ivane Javakhishvili Tbilisi State University, 1 Ilia Chavchavadze Avenue, Tbilisi, 0179, Georgia.

[4]Tbilisi State Medical University, 33 Vazha Pshavela Ave, Tbilisi, Georgia.

[5]M. Iashvili Children's Central Hospital, Lubliana str 13/6, Tbilisi, 0159, Georgia.

[6]Department of Physiotherapy, University Hospitals NHS Foundation Trust, Solihull Hospital, Solihull, B91 2JL, UK.

[7]Community Cardio-Respiratory Clinical Lead, iCares Directorate, Sandwell and West Birmingham NHS Trust, West Bromwich B71 4HJ, UK

[8]Institute of Applied Health Research, University of Birmingham, Birmingham, B15 2TT, UK

[9]Department of General Practice, Peking University First Hospital, Beijing, China

[10]Lung Function & Sleep, Queen Elizabeth Hospital, Birmingham, B15 2TH, UK

[11]International Primary Care Respiratory Group, 19 Armour Mews, Larbert, FK5 4FF, Scotland

[12] Life and Health Sciences Research Institute, School of Medicine, University of Minho. ICVS/3B's, PT Government Associate Laboratory, Braga/Guimarães, 4710-057, Portugal

[13]Health Services Management Centre, School of Social Policy, University of Birmingham, Birmingham, B15 2TT, UK

[14]Family Medicine, ABC Medical School, Sao Paolo, Brazil

[15]NIHR Birmingham Biomedical Research Centre, University Hospitals Birmingham NHS Foundation Trust and University of Birmingham, Birmingham, B15 2TT, UK

For peer review only - http://bmjopen.bmj.com/site/about/guidelines.xhtml

[16]Centre for Family Medicine, Medical Faculty, Skopje, Macedonia

[17]Faculty of Medicine, University of São Paulo, 05403-000, Brazil

For peer review only

For peer review only - http://bmjopen.bmj.com/site/about/guidelines.xhtml

ABSTRACT

Objectives: To assess the feasibility of delivering a culturally tailored pulmonary rehabilitation programme and conducting a definitive randomised controlled trial.

Design: A two-arm, randomised feasibility trial with a mixed-methods process evaluation.

Setting: Secondary care setting in Georgia, Europe

Participants: People with symptomatic spirometry confirmed COPD recruited from primary and secondary care.

Interventions: Participants were randomised in a 1:1 ratio to a control group or intervention comprising 16 twice-weekly group pulmonary rehabilitation sessions tailored to the Georgian setting.

Primary and secondary outcome measures: Feasibility of the intervention *and* RCT were assessed according to: study recruitment, consent and follow-up, intervention fidelity, adherence and acceptability, using questionnaires and measurements at baseline, programme-end and 6-months, and through qualitative interviews.

Results: The study recruited 60 participants (as planned): 54 (90%) were male, 37 (62%) had an FEV1% predicted of ≤50%. The mean MRC score was 3.3 (SD 0.5), and mean SGRQ 50.9 (SD 17.6). The rehabilitation specialists delivered the pulmonary rehabilitation with fidelity. Thirteen (43.0%) of participants attended at least 75% of the 16 planned sessions. Participants and rehabilitation specialists in the qualitative interviews reported that the programme was acceptable, but drop-out rates were high in participants who lived outside Tbilisi and had large distances to travel. Outcome data was collected on 63.3% at 8 weeks and 88.0% at 6 months. Mean change in SGRQ total was -24.9 [95% CI -40.3, -9.6] at programme end and -4.4 [95% CI -12.3, 3.4] at 6 months follow-up for the intervention group and -0.5 [-8.1, 7.0] and -8.1 [-16.5, 0.3] for the usual care group at programme end and 6 months respectively.

Conclusions: The tailored pulmonary rehabilitation intervention was feasible to deliver. Approaches to improve uptake and adherence warrant further research.

Trial registration: ISRCTN16184185

For peer review only - http://bmjopen.bmj.com/site/about/guidelines.xhtml

Strengths and limitations of this study

- This is the first published pulmonary rehabilitation trial undertaken in Georgia.
- It is an intervention culturally tailored for a middle income country, one of local importance, having been selected through a structured prioritisation process involving local policy makers, clinicians and patients.
- The low 8-week follow-up rate in the intervention group (63%) makes the findings difficult to interpret at this time point.
- A post-hoc per-protocol analysis explored the outcomes in intervention participants who attended at least 50% of the pulmonary rehabilitation sessions, showing a consistent pattern of improvements
- Recruitment through primary care proved challenging due to patients with COPD diagnosis from primary care not fulfilling diagnostic criteria on spirometry.

For peer review only

INTRODUCTION

Chronic obstructive pulmonary disease (COPD) is a progressive chronic inflammatory lung disease that has a significant health and economic burden, with high health care costs arising largely from hospital admissions for exacerbations.[1,2] It is currently the 4th leading cause of death worldwide[3,4] with 251 million cases reported globally in 2016.[5] Although more than 90% of these deaths occur in low and middle income countries (LMICs),[6] most research on COPD management has been undertaken in high-income countries. The burden of chronic respiratory disease (CRD) is a considerable challenge for Georgia's healthcare system with the mortality attributable to CRD about 4% in Georgia[7], and as elsewhere in the world, rates are increasing mainly due to air pollution (outdoor and indoor from solid fuel cooking and heating;[8,9]) and high rates of tobacco use[7] (about one third of adults are current smokers). The availability of pharmacotherapy is limited due to resource constraints, making pharmacological management difficult.

In addition to the direct health and healthcare burden, COPD negatively impacts the health-related quality of life (HRQoL) of people living with the condition. There is growing evidence that pulmonary rehabilitation (PR) is an effective and cost-effective therapeutic intervention to improve COPD symptoms, patients' quality of life and to reduce risk of premature deaths.[10] However, evidence of the effectiveness of PR for COPD in patients from LMICs is extremely limited and few studies have adapted PR to the local context in LMICs.[11] There are currently no PR services offered to patients in Georgia. In this context, where resources are limited, yet COPD is a major burden, research is needed to evaluate whether offering PR is feasible and to assess whether the intervention would have similar effects to those observed in other settings. This study evaluated the feasibility of a future trial to assess the effectiveness of a culturally tailored PR programme in Georgia.

METHODS

Study design and participants

The study was a two-arm, non-blinded, randomised feasibility trial of an adapted PR programme, with a mixed-methods process evaluation conducted in the Chapidze Emergency Cardiology Center, Tbilisi. Participants with an MRC score ≥2, and a spirometry-confirmed COPD diagnosis, defined as a post-bronchodilator FEV_1/FVC ratio <0.70, were deemed eligible for the study. Exclusion criteria were the presence of musculoskeletal or neurological conditions preventing exercise, unstable cardiovascular

For peer review only - http://bmjopen.bmj.com/site/about/guidelines.xhtml

disease (e.g. unstable angina, aortic valve disease, unstable pulmonary hypertension), severe cognitive impairment, severe psychotic disturbance, active contagious infectious disease, and very poor balance.

Sample size

We aimed to recruit 60 participants. The sample size was chosen to enable estimation of feasibility outcomes with reasonable precision.[12] A follow-up rate of 80% could be estimated with a precision of 68% to 89% (binomial exact 95% confidence interval).

Recruitment and randomisation

Participants were recruited (December 2018- May 2019) from six primary care facilities and three secondary care facilities in Tbilisi; all facilities provided services to patients without geographical restriction. An information brochure about the programme was distributed among doctors in primary care facilities and doctors from the Georgian Respiratory Association working in secondary healthcare. These facilities provided contact details of COPD patients, who were contacted via telephone. The MRC score was completed over the telephone and those with a score of ≥2 were invited to the spirometry eligibility assessment.[13] Before spirometry, patients were given a study participant information leaflet. Post-bronchodilator spirometry was performed according to American Thoracic Society and European Respiratory Society 2005 guidelines[14] using a Spirolab III spirometer and over-read by a member of the research team (AE or BC). Eligible participants were asked to give informed, written consent to participate in all aspects of the trial, including qualitative interviews. Randomisation to one of the two groups in a 1:1 ratio was performed using an electronic database (REDcap).[15]

Intervention

The intervention was adapted from PR programmes delivered in the UK.[16] Three focus groups took place in Tbilisi, Georgia and involved people with COPD (n=6), family practitioners and respiratory physicians (n=6), and rehabilitation specialists (n=7) who discussed potential components for the PR programme. Their views were considered alongside the APEASE criteria[17] (Affordability, Practicality, Effectiveness and cost-effectiveness, Acceptability, Side-effects/Safety, Equity). Adaptations were made to the programme, taking into consideration contextual, cultural and resource needs in Georgia. Given the high literacy rates of the population, an information booklet about COPD, covering the 16 topics included in the PR education sessions, was also produced in the local language. The main adaptations included recognition of the importance of including family members, by inviting them to attend a pre-

For peer review only - http://bmjopen.bmj.com/site/about/guidelines.xhtml

rehabilitation information session and the educational talks. The educational sessions did not include discussions of end of life or the potentially terminal nature of COPD as requested by focus group participants.

A pre-rehabilitation one-to-one assessment was booked at a time convenient for the patient. The aim of this assessment was to orientate the patient to the PR intervention and tailor their exercise programme. During this visit, patients were given a pulmonary rehabilitation information booklet, created for the trial by the research team. The distance walked in the incremental shuttle walk test (ISWT)[18], Borg rating of perceived exertion and modified Borg breathlessness scales[19] were used to prescribe the intensity of the exercise component of the PR programme. Participants had pulse-oximetry measured during the ISWT and were prescribed 65 to 85% of their maximal baseline ISWT performance (lower in deconditioned patients).

The adapted PR programme drew on international guidance[16,20] and consisted of two main components: 1½ hours of exercise which took place in a hospital rehabilitation gym and 15-20 minutes of education on the management of COPD covering 16 topics (see Supplementary Table S1 for additional information). The description, content and frequency of these components were determined during the intervention development phase of this project (cultural tailoring). The programme took place twice weekly for an eight week period as a rolling programme (i.e. new patients were introduced at any point).

The intervention was delivered by two rehabilitation specialists, who had not previously delivered PR. They were trained by UK respiratory physiotherapists to deliver PR over two visits and received a total of four days of training. Training topics are detailed in Supplementary Table S2.

Usual care group

Participants randomised to the control group received usual care from their primary care doctor and or pulmonologist. They were offered a delayed 1.5 - 2 hour educational session delivered by pulmonologists and specialists once the final (6-months) follow-up was complete. During this session, participants received a PR educational booklet.

Outcome measures

Data were collected from patients by the research team members at the end of the PR programme (eight weeks) and at six months, at a clinical assessment; participants who were unable or unwilling to

For peer review only - http://bmjopen.bmj.com/site/about/guidelines.xhtml

attend the assessment were invited to complete the questionnaires over the telephone. A baseline questionnaire captured sociodemographic and health-related characteristics of the participants.

Feasibility outcomes

The main feasibility outcomes were delivery of the PR with fidelity, acceptability to participants, recruitment rate, follow-up rate at six months, adherence to the intervention, ability to carry out trial procedures, feasibility of methods to measure costs of PR, other COPD-related health care utilisation (including number of hospital admissions since enrolment) and patient-incurred costs.

Secondary outcomes

Secondary outcomes included the primary outcome of a future definitive RCT, which was the St George's Respiratory Questionnaire (SGRQ)[21] at six months (total, symptoms, activity and impact). Other secondary outcome measures at eight weeks and six months were: SGRQ at programme end, exercise capacity measured by the ISWT (metres),[18] smoking status validated by cotinine, COPD Assessment Test (CAT),[22] self-reported physical activity (IPAQ),[23] self-efficacy measured by the Stanford Self-Efficacy scale,[24] anxiety (PHQ-9)[25] depression GAD-7),[26] and self-reported number of exacerbations (in the last six months).

To assess the feasibility of collecting outcome data for a future health economic analysis we collected the EQ-5D-5L[27], self-reported COPD related health care utilisation and participant incurred costs from an open question in the eight week questionnaire. In order to determine whether it would be possible to estimate the cost of PR in a future trial we recorded the number of sessions attended by each participant and total number of sessions delivered.

Process evaluation

Process measures to determine the feasibility of intervention delivery and study processes included number of PR sessions attended, fidelity of delivery, and participant engagement assessed by observation of 16 sessions. Completion of PR was defined as attendance at 75% of sessions.[28]

One rehabilitation session was observed by a member of the research team and exercise sheets were analysed to make sure they were delivered with fidelity. Fidelity was defined as evidence of increasing exercise prescriptions and recorded activities over the PR course for three aerobic and three strength exercises.

For peer review only - http://bmjopen.bmj.com/site/about/guidelines.xhtml

We telephoned patients who had agreed to be contacted for interview, using purposive sampling to increase the mix of socio-demographic characteristics, severity of COPD and differing levels of engagement with the PR programme. After completion of the programme, semi-structured interviews were conducted with nine participants (who attended between two and sixteen PR sessions) and the two rehabilitation specialists to assess their experience of it. Participant interviews were conducted by telephone, and face to face with the rehabilitation specialists. Structured topic guides (see supplementary table S4) were used and refined iteratively as themes emerged. The average interview duration was 35 minutes for the participants and 33 minutes for the rehabilitation specialists. Sample size was determined by thematic saturation.[29]

Data analysis

Analyses concentrated on available data only, with no attempt made to impute missing values. Analyses commenced once the last participant completed follow up. Participant demographics were tabulated to understand the population recruited and whether recruitment reflected the socio-demographic profile of people with COPD in Georgia. We report recruitment and follow-up rates, with 95% confidence intervals, overall and by study arm. Although the trial was not powered to detect a difference between intervention and wait-list control, we calculated the mean change in SGRQ at PR end and 6 months for those allocated to each group; 95% confidence intervals were provided for estimates obtained. We provided estimates of the secondary outcomes by group and display binary outcomes using counts and percentages, and continuous data are presented using means and standard deviations. Since the study was not powered to detect treatment effects on clinical outcomes, p values and 95% CIs were not reported. Data were analysed using STATA/IC 15.1.[30]

To explore whether the lack of effect at six months in the intervention group was due to lack of sustained effect or lack of engagement with PR by many of the intervention participants a post-hoc per protocol analysis was undertaken. Outcomes of intervention group participants who attended at least 50% of sessions[31] (n=13) were calculated.

Audio recordings of qualitative interviews were transcribed intelligent verbatim, anonymised and analysed using content analysis.[32]

For peer review only - http://bmjopen.bmj.com/site/about/guidelines.xhtml

Patient and public involvement

A research prioritisation exercise was initially conducted with patients, clinicians and policy makers; the need to find effective, affordable COPD management that suited Georgian priorities and processes was selected. A patient advisory group in the UK advised on PR design, highlighting the importance of sessions being local with minimal travel costs. The intervention adaptation had input from six patients who had COPD. The study was overseen by a Trial Steering Committee, which included a person with COPD, who was involved throughout all stages of the study, contributing to decisions by commenting on intervention process, materials, advising on recruitment approaches and dissemination of findings.

RESULTS

Feasibility of conducting a definitive RCT

Participant recruitment and follow-up

We contacted 312 patients in order to recruit 60 for the study. After meeting primary criteria for study inclusion, 159 patients underwent spirometry, of whom 74 had COPD confirmed. Sixty-three patients were assessed for eligibility and attended the baseline visit, 60 were recruited and randomised (30 intervention and 30 usual care) (figure 1). One participant withdrew from the intervention group; follow-up at six months was 88% across both groups. Recruitment from primary care was challenging with the majority (n=53, 88.3%) of participants coming from a hospital setting. Participants who were identified in primary care and assessed for eligibility were frequently excluded following spirometry.

Baseline characteristics of participants

Participant characteristics are detailed in Table 1. Overall, 90% of participants were male, the mean age was 65 years (SD 7.95); most were Georgian (n=56; 93.3%). Twenty-three (38.3%) were current smokers and all had at least one comorbidity. Only 20 (33.3%) were in paid work. Few participants (n=10, 16.7%) were not using any COPD medication. The mean (SD) MRC score was 3.28 (0.52) and 37 (61.7%) had an FEV1% predicted of ≤50% indicating severe COPD. The mean SGRQ was 50.87 (SD 17.59).

For peer review only - http://bmjopen.bmj.com/site/about/guidelines.xhtml

Table 1: Baseline characteristics of the patients for intervention and usual care group.

Characteristic	Intervention N=30	Usual care N=30	All N=60
Sex, n (%) Male	26 (86.7%)	28 (93.3%)	54 (90.0%)
Age in years, mean (SD)	64 (7.3)	65 (8.7)	65 (8)
Ethnicity, n (%)			
Georgian	28 (93.3)	28 (93.3)	56 (93.3%)
Other ethnic group	2 (6.7)	2 (6.7)	4 (6.7%)
Education, n (%)			
No formal qualification	0 (0.0)	2 (6.67)	2 (3.3)
High school education	7 (23.3)	6 (20.0)	13 (21.7)
Degree level or higher	15 (50.0)	14 (46.7)	29 (48.3)
Other	8 (26.7)	8 (26.7)	16 (26.7)
Living alone, n(%)	3 (10.0)	4 (13.3)	7 (11.7)
Relationship status. n (%)			
Married and living with partner	22 (73.3)	22 (73.3)	4 (73.3)
Never married	3 (10.0)	4 (13.3)	7(11.67)
Separated, but still legally married	1 (3.3)	1 (3.3)	2 (3.3)
Divorced	4 (13.3)	1 (3.3)	5 (8.3)
Widowed	0 (0.0)	2 (6.67)	2 (3.3)
Employment status, n (%)			
In paid work (full or part-time including self-employed)	7 (23.3)	13 (43.3)	20 (33.3)
Unemployed/looking for work	10 (33.3)	2 (6.7)	12 (20.0)
Retired from paid work	11 (36.7)	11 (36.7)	22 (36.7)
Looking after the family or home	10 (33.3)	6 (20.0)	16 (26.7)
Unable to work because of my chest/lung problem	5 (16.7)	1 (3.3)	6 (10.0)
Unable to work because of other long term health problem	2 (6.7)	1 (3.3)	3 (5.0)

For peer review only - http://bmjopen.bmj.com/site/about/guidelines.xhtml

Other	6 (20.0)	5 (16.7)	11(18.3)
Co-morbidities – ever diagnosed, n(%)			
Diabetes	5 (16.7)	2 (6.7)	7 (11.7)
High blood pressure	11 (36.7)	11 (36.7)	22 (36.7)
Coronary heart disease/Angina/Heart Attack	6 (20.0)	4 (13.3)	10 (16.7)
Heart failure	7 (23.3)	7 (23.3)	14 (23.3)
Stroke/mini-stroke	0 (0.0)	4 (13.3)	4 (6.7)
Asthma	7 (23.3)	5 (16.7)	12 (20.0)
Tuberculosis	4 (13.3)	2 (6.7)	6 (10.0)
Osteoarthritis	5 (16.7)	4 (13.3)	9 (15.0)
Osteoporosis	1 (3.3)	1 (3.3)	2 (3.3)
Depression	2 (6.7)	2 (6.7)	4 (6.7)
Other condition	5 (16.7)	5 (16.7)	10 (16.7)
Medication use			
SABA/SAMA	9 (30.0)	6 (20.0)	15 (25.0)
LABA/LAMA	10 (33.3)	15 (50.0)	25 (41.7)
ICS	2 (6.7)	2 (6.7)	4 (6.7)
ICS/LABA	15 (50.0)	17 (56.7)	32 (53.3)
Steroids	2 (6.7)	1 (3.3)	3 (5.0)
Methylxanthine	2 (3.3)	2 (3.3)	4 (6.7)
Other	9 (30.0)	9 (30.0)	18 (30.0)
None of the above	6 (20.0)	4 (13.3)	10 (16.7)
Smoking status n (%)			
Current	10 (23.3)	13 (43.3)	23 (38.3)
Ex-smoker	22 (73.4)	21 (70.0)	43 (71.7)
Never smoker	2 (6.67)	2 (6.7)	4 (6.67)
GOLD stage, n (%)			
IV (FEV_1 <30% predicted)	1 (3.3)	1 (3.3)	2 (3.3)

For peer review only - http://bmjopen.bmj.com/site/about/guidelines.xhtml

III (FEV_1 30-49% predicted)	4 (13.3)	4 (13.3)	8 (13.3)
II (FEV_1 50-79% predicted)	19 (63.3)	18 (60.0)	37 (61.7)
I (FEV_1 ≥80% predicted)	6 (20.0)	7 (23.3)	13 (21.7)
MRC Score, mean, (SD)	3.4 (0.6)	3.2 (0.4)	3.3 (0.5)
MRC score 3, n (%)	21 (70.0)	24 (80.0)	45 (75.0)
MRC score 4, n (%)	7 (23.3)	6 (20.0)	13 (21.7)
MRC score 5, n (%)	2 (6.7)	0 (0.0)	2 (3.3)
COPD Assessment Test (CAT) score, mean (SD)	20.3 (5.9)	19.9 (6.6)	20.1 (6.2)
PHQ-9 depression score, mean (SD)	6.2 (4.0)	5.3 (4.1)	5.8 (4.0)
GAD-7 anxiety score, mean (SD)	3.5 (3.2)	3.3 (2.8)	3.4 (3.0)
Stanford Self Efficacy Score, mean (SD)	5.7 (1.9)	6.7 (1.5)	6.2 (1.7)
ISWT, mean (SD)	229.7 (112.7)	249.3 (96.2)	239.5 (104.3)
Self-reported number of exacerbations in the last 6 months, mean (SD)	2.7 (0.9)	2.3 (1.1)	2.5 (1.1)
St Georges Respiratory Questionnaire (SGRQ)			
SGRQ - Impact, mean (SD)	42.0 (19.9)	37.0 (20.3)	39.5 (20.1)
SGRQ - Activity, mean (SD)	67.0 (20.2)	56.9 (24.1)	62.0 (22.6)
SGRQ - Symptoms, mean (SD)	64.2 (17.1)	65.4 (15.0)	64.8 (16.0)
SGRQ - Total, mean (SD)	53.6 (17.2)	48.1 (17.9)	50.9 (17.6)
Total Physical Activity Scores, MET-minutes/week (SD)	2092.8 (2647.2)	3027.7 (3303.9)	2560.3 (3005.3)

FEV1 – forced expiratory volume in 1 second; GAD: generalized anxiety Disorder-7; ICS: inhaled corticosteroid; LABA: long-acting beta agonist; LAMA: long-acting muscarinic antagonist; MET: metabolic equivalent of task; PHQ9: Patient Health Questionnaire-9; SABA: short-acting beta agonist; SAMA: short-acting muscarinic antagonist; SD: standard deviation.

Follow-up rates

Follow up rates at eight weeks were 63.3% (38); but were much lower in the intervention arm (13; 43.3%) compared to the usual care group (25; 83.3%). Participants who did not complete follow-up at eight weeks had similar characteristics to the full sample and did not differ by smoking status, SGRQ or

For peer review only - http://bmjopen.bmj.com/site/about/guidelines.xhtml

PHQ9 score. Follow-up at 6 months was 88.3% (n=53); seven participants were administered the questionnaire by telephone, so did not undertake the ISWT, hand grip strength or cotinine measurement. One participant completed the questionnaire by telephone but attended the assessments.

Completeness of follow-up questionnaires

The level of completion of the questionnaire was high in all cases where the questionnaire was completed at the assessment and over the phone with 100% completion of questions.

Feasibility of economic evaluation in definitive RCT

The EQ-5D-5L and self-reported COPD-related health care utilisation questions had high levels of completion. Intervention participants reported costs mainly related to transportation (bus, petrol fuel, taxi). The cost of the programme came up as an important determinant for attendance in the future. Most participants noted that they would not be able to attend PR if they had to pay for it. A small number noted they would be able to pay 15-20 euros or 10% of the fee. Equipment costs, number of sessions delivered and staff costs for PR session delivery were established; the component costs are detailed in Supplementary Table S3.

Outcomes of a future RCT

At programme end (eight weeks), those with follow-up data in the intervention group (n=13) showed substantial improvements across all the outcomes including the SGRQ and ISWT, with mean changes (SD) of -24.9 (25.4) [95%CI -40.3, -9.6] and 120.8m (89.5) respectively. The usual care group (n=26) showed only minimal changes (Table 2). At the six month follow-up improvements in the intervention (n=27) were still visible, but much more modest. The usual care group (showed considerable improvements at the six month follow-up for all the outcomes except for the ISWT and exacerbations (Table 2).

For peer review only - http://bmjopen.bmj.com/site/about/guidelines.xhtml

Table 2: Effect of pulmonary rehabilitation at 8 weeks and 6 months for patients in the intervention and usual care group

	**8 week follow-up**				**6 month Follow-up**			
	**Intervention**		**Usual care**		**Intervention**		**Usual care**	
	**Mean (SD) N=13**	**Mean change (SD) [95% CI]**	**Mean (SD) N=25**	**Mean change (SD) [95% CI]**	**Mean (SD) N=27**	**Mean change from baseline (SD) [95% CI]**	**Mean (SD) N=26**	**Mean change from baseline (SD) [95% CI]**
SGRQ - Total[a]	32.3 (27.4)	-24.9 (25.4) [-40.3, -9.6]	48.0 (20.8)	-0.5 (18.0) [-8.1, 7.0]	48.1 (23.5)	-4.4 (19.8) [-12.3, 3.4]	40.8 (20.0)	-8.1 (20.8) [-16.5, 0.3]
SGRQ - Impacts	23.3 (25.6)	-22.7 (22.6) [-36.4, -9.1]	37.6 (23.2)	0.3 (22.9) [-9.2, 9.7]	38.4 (23.6)	-2.4 (21.0) [-10.7, 5.9]	30.1 (20.0)	-7.5 (25.5) [-17.7, 2.9]
SGRQ - Activity	42.5 (32.9)	-30.5 (29.4) [-48.3, -12.8]	58.6 (20.3)	1.1 (17.7) [-6.2, 8.5]	58.5 (29.8)	-7.8 (26.5) [-18.3, 2.7]	49.2 (24.8)	-10.5 (23.3) [-19.9, -1.1]
SGRQ - Symptoms	41.0 (31.6)	-21.6 (37.3) [-44.1, 1.0]	60.5 (25.7)	-4.2 (21.4) [-13.2, 4.9]	58.1 (23.4)	-4.5 (24.1) [-14.1, 5.0]	57.7 (24.8)	-6.1 (22.4) [-15.1, 3.1]
MRC Score	2.5 (1.2)	-0.9 (1.3)	3.0 (0.6)	-0.2 (0.6)	2.9 (1.0)	-0.4 (1.0)	2.8 (0.9)	-0.3 (0.8)
COPD Assessment Test (CAT) score	13.8 (8.5)	-6.6 (7.4)	18.3 (7.0)	-1.4 (5.0)	17.9 (8.5)	-1.7 (7.3)	17.7 (7.6)	-2.7 (6.5)
PHQ9 depression score	2.8 (1.9)	-2.8 (2.9)	5.5 (5.1)	-0.3 (3.9)	4.74 (4.2)	-1.2 (3.8)	3.8 (3.0)	-1.9 (3.2)
GAD anxiety score	1.9 (1.6)	-0.9 (3.0)	3.3 (3.5)	-0.3 (2.8)	2.51 (3.3)	-0.7 (2.8)	2.0 (2.1)	-1.3 (2.6)
Stanford Self Efficacy Score	7.8 (1.2)	1.9 (1.5)	6.9 (1.5)	0.3 (1.8)	7.0 (1.5)	1.2 (2.1)	7.3 (1.2)	0.7 (1.5)
ISWT	316.2 (134.3)	120.8 (89.5)	247.6 (113.5)	-13.2 (110.7)	217.6 (89.6)	-7.1 (92.5)	216 (99.4)	-29.2 (74.1)
Number of exacerbations in the last 6 months	3.5 (1.1)	0.7 (1.3)	3.6 (1.0)	1.3 (1.7)	3.2 (1.3)	0.5 (2.5)	3.2 (1.3)	0.92 (1.7)

For peer review only - http://bmjopen.bmj.com/site/about/guidelines.xhtml

Total Physical Activity Scores. MET-minutes/week (SD)	783.2 (1509.9)	-1309.6 (2539.0)	1634.7 (1532.0)	-1393.1 (2838.3)	943.6 (1163.5)	-1653.3 (4650.6)	1857.0 (2853.4)	-2386.7 (5537.9)

[a]A reduction in SGRQ is a positive patient outcome

Outcomes in relation to smoking are reported in Table 3. At the 8-week follow-up none of the participants had quit smoking, but two of the intervention and three of the usual care group had made quit attempts. By the 6-month follow-up, one of the intervention group and two of the usual care group had quit smoking and an additional four of each group had made quit attempts.

Table 3: Self-reported tobacco use at 8 weeks and 6 months

	**8 week FU**		**6 month FU**	
**Tobacco use**	**Intervention N=13 n (%)**	**Usual care N=25 n (%)**	**Intervention N=27 n (%)**	**Usual care N=26 n (%)**
Current smoker at study start	3 (23.1%)	10 (40.0%)	9 (33.3%)	9 (34.6%)
Current smoker at follow-up	3 (23.1%)	8 (32.0%)	10 (37.0%)	7 (26.9%)
Smokers at baseline who tried to quit since enrolling in the research study	2 (66.7%)	3 (30.0%)	4 (44.4%)	4 (44.4%)
Smokers at baseline who quit since enrolling in the trial (cotinine validated)	0 (0%)	1 (10.0%)	1 (11.1%)	2 (22.2%)

Attendance and adherence

Eight participants attended all 16 sessions, 13 (43.3%; or 54.2% of those who attended at least one session) attended at least 75% and six did not attend any sessions (supplementary figure 1). Participants gave several reasons for not attending PR sessions particularly highlighting “cold weather, family issues and not being in town”.

For peer review only - http://bmjopen.bmj.com/site/about/guidelines.xhtml

Per-protocol analysis

At the six month assessment only 13 intervention participants who had completed 8 (50%) or more PR sessions were followed up. Table 4 reports the per-protocol analysis at 6 months; the 8-week results are in supplementary table S5.

The SGRQ would be the primary outcome of a future RCT and at 6-months large, sustained improvements were seen across all the domains for the intervention group. A consistent pattern of sustained improvements were seen across all the outcomes, apart from the ISWT which showed a modest improvement at 6-months.

For peer review only

Table 4: Post-hoc per-protocol analysis: 6 month follow-up of intervention patients who attended 50% or more pulmonary rehabilitation sessions compared to usual care

	Intervention n=13		Usual care n=26	
	**Mean (SD)**	**Mean change (SD) [95% CI]**	**Mean (SD)**	**Mean change (SD) [95% CI]**
SGRQ - Total	44.9 (24.3)	-16.6 (14.5) [-25.3, -7.8]	40.8 (20.0)	-8.1 (20.8) [-16.5, 0.3]
SGRQ - Impacts	36.6 (22.7)	-14.1 (16.8) [-24.3, -4.0]	30.1 (20.0)	-7.5 (25.5) [-17.7, 2.9]
SGRQ - Activity	53.3 (33.7)	-22.9 (21.3) [-35.8, -10.1]	49.2 (24.8)	-10.5 (23.3) [-19.9, -1.1]
SGRQ - Symptoms	54.7 (24.8)	-12.6 (20.1) [-24.7, -0.4]	57.7 (24.8)	-6.0 (22.4) [15.1, 3.1]
MRC score	2.6 (1.0)	-1.0 (0.8)	2.8 (0.9)	-0.3 (0.8)
CAT	16.2 (7.1)	-5.5 (4.8)	17.7 (7.6)	-2.7 (6.5)
PHQ-9 depression score	4.8 (5.0)	-0.9 (3.4)	3.8 (3.0)	-1.9 (3.2)
GAD anxiety score	2.8 (3.6)	-0.8 (3.2)	2.0 (2.1)	-1.3 (2.6)
Stanford Self Efficacy Score	7.3 (1.7)	1.6 (2.0)	7.3 (1.2)	0.7 (1.5)
ISWT	217.5 (85.7)	17.5 (72.6)	216 (99.4)	-29.2 (74.1)
Self-reported number of exacerbations in the last 6 months	2.9 (1.5)	0.1 (1.5)	3.2 (1.27)	0.9 (1.7)
Total Physical Activity Scores. MET-minutes/week	1078.5 (1498.5)	72.92 (1816.8)	1857.0 (2853.4)	-1170.7 (2753.3)

For peer review only - http://bmjopen.bmj.com/site/about/guidelines.xhtml

Intervention fidelity

Observation of one rehabilitation session and assessment of the rehabilitation specialists' records identified high levels of fidelity with the intervention delivery. In all participants there was an increase in numbers of repetitions/intensity of at least some of the exercises over their participation period.

> *"…their exercise intensity is increasing, number of sets is increasing," (Rehab specialist)*

Intervention acceptability

Most patients had a positive experience with the exercise, specialists, educational sessions and the booklet. Education sessions and guidance by the rehabilitation specialists were highly valued.

Most patients felt satisfied with the programme; some mentioned they felt it did not influence their behaviour much, whilst some emphasised that they felt more "joyful." Transport, hot weather during summer, health, money and work-related reasons were mentioned as barriers to continuing with the PR programme. Overall, the exercises were well received but suggestions to include more breathing exercises were made.

> *"[my physical activity] has changed, I walk more freely. When I need to lift heavy things, it is not difficult for me. It worked very well on me, and psychologically I am very well disposed" (P2)*

> *Barriers: "weather and my health" (P4)*

> *"I missed last two sessions … Transportation was a challenge." (P5)*

The rehabilitation specialists reported that many participants improved and that attending the rehabilitation in a group increased their motivation.

> *"Those who completed 16 exercise sessions have asked us to print out exercise sheets. Some of the patients who had completed the exercises asked us to continue their exercises here, since the atmosphere here was different and group exercise was increasing their motivation." (Rehab specialist)*

> *"Many patients did not know how to manage breathing, how to go outside confidently, did not know how to manage sexual relations, nutrition, what would be beneficial and what was not necessary. COPD patients usually are thin, because of their disease. Due to physical activity, one*

For peer review only - http://bmjopen.bmj.com/site/about/guidelines.xhtml

patient gained 7kg and another one 5kg, which made them very happy and they say result of the exercises." (Rehab specialist)

However, for some participants motivation was an issue as well as barriers of travel and time.

"but patients were missing sessions nevertheless. Those who had money, did not have time, and some did not work and transportation was a problem." (Rehab specialist)

Adverse events

There were no adverse events reported throughout the study.

DISCUSSION

This study aimed to determine the feasibility of delivering a culturally tailored PR programme in Georgia. Our results indicate that it was feasible to train rehabilitation specialists to deliver the PR with fidelity. There were challenges in recruiting participants from primary care, as many general practitioners do not have access to spirometry within the primary care setting. As a consequence, many patients assessed from primary care did not have an objective measure to support their diagnosis and on spirometry did not fulfil the criteria for COPD. Adherence to the programme was sub-optimal with only 50% of intervention group participants attending at least 50% of the planned sessions. The programme was acceptable to the participants who attended, but clearly the fact that 20% of intervention participants did not attend any sessions suggests that there were considerable barriers to attendance. The rehabilitation specialists reported high drop-out rates in participants who lived outside Tbilisi and had long journey times (<1 hour). Participants who attended demonstrated improvements in clinical outcomes. In a post-hoc per-protocol analysis of intervention group participants who attended at least 50% of planned sessions, the outcomes were well sustained to six months.

The Cochrane review of PR for COPD[33] reported significant improvements in all the domains of the Chronic Respiratory Questionnaire and for the SGRQ, with the total score improving more than the minimally clinically important difference of four points, which was mirrored in our study. Of the 65 RCTs included the majority were from high income settings and many of those from LMICs had small sample sizes.

For peer review only - http://bmjopen.bmj.com/site/about/guidelines.xhtml

We undertook some cultural tailoring of the PR before implementation, but did not find a need to make major changes to the programme given the delivery context in a secondary care setting. However, the discussion of end of life care was deemed unacceptable by participants from all three focus groups during the cultural adaptation, so was not included. Other researchers[34] have made significant adaptations to PR for patients who have recovered from tuberculosis, in Uganda, reporting high levels of engagement and good outcomes,[34] and in Greece,[28] and evaluated implementation in non-randomised studies.

Strengths and limitations

This study has a number of strengths. Whilst there have been many trials of PR, there are no published findings of trials undertaken in the Georgian setting. The topic was selected following a structured prioritisation process involving policy makers, clinicians and patients in Tbilisi, Georgia.[35] We undertook some cultural tailoring prior to finalising the final form of PR to be delivered to increase acceptability. The training was delivered by experienced trainers from the UK.

The study also has some limitations. A key issue was the low follow-up rate at end of programme in the intervention group resulting in an overall follow-up rate of 63% at eight weeks; similar to results reported in other studies.[36,37] The differential follow-up at 8-weeks makes the findings difficult to interpret. The fall-off in effects between eight weeks and six months in the intervention group might have been the result of more adherent participants attending the 8-week follow-up. To address this issue our post-hoc per protocol analysis explored the effects of the intervention in those participants most adherent to the PR programme and identified sustained improvements in health outcomes in this group. We faced challenges in recruiting primary care patients with COPD, largely due to the lack of availability of spirometry for diagnosis.[38] This could be overcome by alternatives to spirometry such as peak flow and questionnaires to diagnose COPD.[39] Studies from other settings have also reported structural and health care practitioner factors affecting the quality of care delivered for COPD.[40-42]

In our study only 43% would categorized as having completed the programme, i.e. attended at least 75% of the planned sessions. Adherence to PR is variable with reported non-completion rates from 42% across a range of high income settings[43] to 38% in the UK in 2017.[44] Previous PR research has reported similar barriers to our study, including transportation difficulties, financial barriers, lack of support, illness and lack of motivation.[45] The literature reports large drop-out rates between referral and pre-assessment and up to 15% of participants not continuing to PR following the pre-assessment.[44,47] We

For peer review only - http://bmjopen.bmj.com/site/about/guidelines.xhtml

randomised participants prior to the pre-rehabilitation session and 20% of our intervention participants did not commence the PR programme; other researchers have reported high levels of non-attendance with up to 50% of people referred to PR not attending a single session.[456]

Our study findings may not apply to patients with COPD managed in primary care in Georgia, as 89% of our study participants were recruited from secondary care and therefore had more severe disease. If PR is implemented in Georgia, referrals will either need to be from secondary care, or spirometry or other diagnostic methods[39] included in the referral pathway from primary care, or inclusion criteria for PR widened to include those without confirmed COPD.

Our population may not be representative of all those with COPD in Georgia as a high proportion of participants were from secondary care, had considerable symptoms (all MRC score 3 or more), yet 16.7% were not taking medication for their lung condition.

Recommendations for future research

This study highlighted deficiencies in COPD diagnosis in primary care in Georgia and future research could explore ways to strengthen management of CRDs outside the secondary care setting.

A large trial or implementation research in the Georgian context would establish optimal delivery and effectiveness of PR for COPD. Additionally, the effectiveness of PR in Georgia for patients with other respiratory diseases and post-COVID lung damage might be undertaken.[48-50] The decision about progression to a full trial or an implementation study should draw on the feasibility outcomes of findings. Our low PR completion rate suggests that some additional work is needed to address access and acceptability. We were able to recruit participants, achieving our planned sample size; the intervention was implemented with acceptable fidelity (defined as education components delivered and exercise delivered to planned intensity), and at 6-months we achieved 88% follow up for the proposed primary outcome of a definitive trial. However, defining programme completion as 75% session attendance, only 43% of the intervention group achieved this. Given the very minor adaptations made to the PR programme we would argue that future research should focus on best methods of implementation rather than effectiveness.

For future intervention delivery, the issue of accessibility needs to be considered, with rehabilitation provision near to where people live. The PR programme was delivered in a secondary care setting in

For peer review only - http://bmjopen.bmj.com/site/about/guidelines.xhtml

Georgia and consideration should be given to delivery in community settings, or even home-based programmes. These could draw on the remote[51] and home-based programmes delivered in response to the COVID-19 pandemic.[52] Widespread roll-out would require training of the rehabilitation specialist workforce in Georgia. Another significant barrier is cost, as PR is not currently included within the free health care provision in Georgia. However, since this research was prioritized by policy makers in Georgia there is a clear recognition that it may be valuable, offering a possible route to change this in the future.

Conclusions

These findings demonstrate the feasibility of PR delivery in Georgia and showed promising outcomes for those participants who adhered to the intervention. However, investment to enable primary care to diagnose and refer people with COPD is needed. Challenges with retention suggest that future PR programmes need to be delivered close to where people live and possibly provide flexibility in methods of delivery.

Acknowledgements:

We gratefully acknowledge:

The International Primary Care Respiratory Group for introducing us to the primary care networks involved in this study and for its continued facilitation of clinical engagement.

The Trial Steering Group Committee and the International Scientific Advisory Committee: Prof Niels Chavennes, Leiden University (Chair); Dr Semira Manaseki-Holland, University of Birmingham; Dr N. Zhvania (Professor in cardiology, Georgia); Revaz Vachnadze (patient representative) Georgia; Prof. Debbie Jarvis, Imperial College London (Chair); Dr Nega Milevsla, Centre for Regional Policy Research and Cooperation “Studiorum”; Skopje; Gordna Kunovska, Skopje.

We especially want to thank: the patients included in the study, the Chapidize Emergency Cardiology Center for providing the rehabilitation facilities, Kelly Redden-Rowley and Helen Beadle for delivering the PR training, the Georgian rehabilitation specialists Shota Bzarashvili and Teona Kvitsadze for delivering the PR - without all of you this project would not have been possible.

For peer review only - http://bmjopen.bmj.com/site/about/guidelines.xhtml

Finally, we would like to acknowledge Radmila Ristovska (1955-2020), also involved in the initiation of this study.

Contributors: KJ, RJ, PA co-led the study design, with contributions and advice from all other authors. TM was the local PI and oversaw all activities in Georgia. The cultural adaptation was overseen by NM, KJ, KR. The team from Georgia (MM, IK, NM, TM, IC, KG, NC) contributed to study design, day to day trial management, recruitment, intervention and follow up overseen by KJ, RA, KR, AT, RJ, PA. AE oversaw the quality of lung function testing. KRR, HB delivered the PR training to the Georgian rehabilitation specialists. NM, MM collected the qualitative data overseen by KR, RA, NG. AS, SJ designed the analysis plan and economic data collection respectively. IK conducted the statistical analysis, supported by AS, AD, KJ. KJ, MM, NM?, IK wrote the manuscript with input from all other authors. As part of the Breathe Well Global Health Research Group, all authors contributed to the development and oversight of this study. All authors contributed to and approved the final version.

Funding: This research was funded by the National Institute for Health Research (NIHR) (NIHR global group on global COPD in primary care, University of Birmingham, (project reference: 16/137/95) using UK aid from the UK Government to support global health research. KJ is part-funded by the NIHR Applied Research Collaboration West Midlands (ARCWM). The views expressed in this publication are those of the author(s) and not necessarily those of the NIHR, ARCWM or the UK Department of Health and Social Care.

Competing interests: The principal investigators have no competing interests in relation to the study described in this paper.

Patient consent for publication: Not required.

Ethical approval: The study was approved by Acad. G.Chapidze Emergency Cardiology Center, Medical Ethical Committee (session protocol №2-2018) and by the University of Birmingham ERN_18-0856.

Data availability statement: All data are available on request from the corresponding author subject to a data sharing agreement.

For peer review only - http://bmjopen.bmj.com/site/about/guidelines.xhtml

REFERENCES

1. Chronic obstructive pulmonary disease (COPD). World Health Organization (date accessed 22/11/20). URL: https://www.who.int/news-room/fact-sheets/detail/chronic-obstructive-pulmonary-disease-(copd)

2. May SM, Li JT. Burden of chronic obstructive pulmonary disease: healthcare costs and beyond. *Allergy Asthma Proc*. 2015;36(1):4-10 doi:10.2500/aap.2015.36.3812

3. COPD Costs 2019 (date accessed 22/11/20). URL: https://www.cdc.gov/copd/infographics/copd-costs.html

4. World Health Organization. Burden of COPD. URL: https://www.who.int/respiratory/copd/burden/en/ (date accessed 23/11/20).

5. Global initiative for chronic obstructive lung disease. GOLD-2020-REPORT-ver1.1wms.pdf https://goldcopd.org/wp-content/uploads/2019/11/GOLD-2020-REPORT-ver1.1wms.pdf (date accessed 22/11/20).

6. Chronic obstructive pulmonary disease (COPD). World Health Organization (date accessed 22/11/20). URL: https://www.who.int/news-room/fact-sheets/detail/chronic-obstructive-pulmonary-disease-(copd)

7. World Health Organization. Non-communicable diseases RISK FACTORS STEPS research Georgia 2016. https://www.who.int/ncds/surveillance/steps/STEPS_Georgia_2016_GEO_report_2016_final.pdf?ua=1 (date accessed 06/06/21).

8. The Tobacco Atlas, from the American Cancer Society. Georgia Country Facts. (date accessed 22/11/20). URL: https://tobaccoatlas.org/wp-content/uploads/pdf/georgia-country-facts.pdf

9. Gamkrelidze A, Mebonia N, Sturua L, et al. Non-communicable Diseases Risk-factors STEPS survey, Georgia 2016. WH. Available: https://www.who.int/ncds/surveillance/steps/STEPS_Georgia_2016_ENG_summary_2016_final.pdf (date accessed 01/06/2021).

10. The Mayo Clinic from the Mayo Foundation for Medical Education and Research COPD - Symptoms and causes. URL: https://www.mayoclinic.org/diseases-conditions/copd/symptoms-causes/syc-20353679 (date accessed 23/11/20).

11. Habib GMM, Rabinovich R, Divgi K, *et al.* Systematic review of clinical effectiveness, components, and delivery of pulmonary rehabilitation in low-resource settings. *npj Prim Care Respir Med 2020;*30:52. https://doi.org/10.1038/s41533-020-00210-y

12. Lancaster GA, Dodd S, Williamson PR. Design and analysis of pilot studies: recommendations for good practice. *Journal of Evaluation in Clinical Practice* 2004; 10(2):307–12. https://onlinelibrary.wiley.com/doi/abs/10.1111/j..2002.384.doc.x (date accessed 22/11/20).

For peer review only - http://bmjopen.bmj.com/site/about/guidelines.xhtml

13. Gough J. Discussion on the diagnosis of pulmonary emphysema. *Proc R Soc Med* 1952; 45(9):576–86. https://www.ncbi.nlm.nih.gov/pmc/articles/PMC1987525/ (date accessed 22/11/20).

14. Miller MR, Hankinson J, Brusasco V, et al. Standardisation of spirometry. *Eur Respir J* 2005;**26**:319–38.

15. PA Harris, R Taylor, BL Minor, et al. REDCap Consortium, The REDCap consortium: Building an international community of software partners, *J Biomed Inform.* 2019 May 9 doi: 10.1016/j.jbi.2019.103208

16. Bolton CE, Bevan-smith EF, Blakey JD, et al. British Thoracic Society guideline on pulmonary rehabilitation in adults: accredited by NICE. *Thorax* 2013;68:ii1-ii30 doi:10.1136/thoraxjnl-2013-203808

17. Michie S, Atkins L, West R. The Behaviour Change Wheel: A Guide to Designing Interventions. London: Silverback publishing 2014.

18. Singh SJ, Jones PW, Evans R, et al. Minimum clinically important improvement for the incremental shuttle walking test. *Thorax* 2008;**63:**775-777.

19. Muza SR, Silverman MT, Gilmore GC, et al. Comparison of scales used to quantitate the sense of effort to breathe in patients with chronic obstructive pulmonary disease. *Am Rev Respir Dis* 1990;**141**:909–913.

20. Rochester CL, Vogiatzis I, Holland AE, et al. An official American Thoracic Society/European Respiratory Society policy statement: enhancing implementation, use, and delivery of pulmonary rehabilitation. *Am J Respir Crit Care Med* 2015;192(11):1373–1386.

21. Jones PW, Quirk FH, Baveystock CM, et al. A Self-complete Measure of Health Status for Chronic Airflow Limitation: The St. George's Respiratory Questionnaire. *American Review of Respiratory Disease* 2012 Dec 17; https://www.atsjournals.org/doi/pdf/10.1164/ajrccm/145.6.1321 (date accessed 22/11/20).

22. Jones PW, Harding G, Berry P, et al. Development and first validation of the COPD Assessment Test. *European Respiratory Journal* 2009 Sep 1; 34(3):648–54. https://erj.ersjournals.com/content/34/3/648 (date accessed 23/11/20).

23. Craig CL, Marshall AL, Sjöström M, et al. International physical activity questionnaire: 12-country reliability and validity. *Med Sci Sports Exerc* 2003 Aug;35(8):1381–95.

24. Lorig K, Sobel D, Ritter P, et al. Effect of a self-management program on patients with chronic disease. *ECP* 2000;30(4):256–62.

For peer review only - http://bmjopen.bmj.com/site/about/guidelines.xhtml

25. Kroenke K, Spitzer RL, Williams JBW. The PHQ-9. *J Gen Intern Med* 2001 Sep;16(9):606–13. https://www.ncbi.nlm.nih.gov/pmc/articles/PMC1495268/ (date accessed 2/11/20).

26. Spitzer RL, Kroenke K, Williams JBW, Löwe B. A brief measure for assessing generalized anxiety disorder: the GAD-7. *Arch Intern Med* 2006;166(10):1092–7.

27. Herdman M, Gudex C, Lloyd A, et al. Development and preliminary testing of the new five-level version of EQ-5D (EQ-5D-5L). *Qual Life Res* 2011 Dec;20(10):1727–36. https://www.ncbi.nlm.nih.gov/pmc/articles/PMC3220807/ (date accessed 23/11/20).

28. Anastasaki M, Trigoni M, Pantouvaki A, et al. Establishing a pulmonary rehabilitation programme in primary care in Greece: A FRESH AIR implementation study. *Chron Respir Dis* January 2019; 16 doi:10.1177/1479973119882939.

29. Glaser B, Strauss A. *Grounded Theory: The Discovery of Grounded Theory*. New York: de Gruyter 1967.

30. StataCorp. *Stata Statistical Software: Release 15*. College Station, TX: StataCorp LLC 2017.

31. Johnson K, Young M, Grimmer K, et al. Frequency of referral to and attendance at a pulmonary rehabilitation programme amongst patients admitted to a tertiary hospital with chronic obstructive pulmonary disease. *Respirology* 2013;18(7):1089-1094.

32. Hsieh H-F, Shannon SE: Three approaches to qualitative content analysis. *Qual Health Res* 2005;15(9):1277–1288.

33. McCarthy B, Casey D, Devane D, et al. Pulmonary rehabilitation for chronic obstructive pulmonary disease. *Cochrane Database of Systematic Reviews* 2015;(2). https://www.cochranelibrary.com/cdsr/doi/10.1002/14651858.CD003793.pub3/full (date accessed 11/08/20).

34. Jones R, Kirenga BJ, Katagira W, et al. A pre-post intervention study of pulmonary rehabilitation for adults with post-tuberculosis lung disease in Uganda. *Int J Chron Obstruct Pulmon Dis* 2017;11(12):3533-3539. doi:10.2147/COPD.S146659.

35. Dickens A, Adab A, Cheng KK, et al. Using a rapid prioritisation process to identify health research priorities in LMICs. *ERJ* 2018;52:PA905 doi:10.1183/13993003.congress-2018.PA905.

36. Finnerty JP, Keeping I, Bullough I, et al. The effectiveness of outpatient pulmonary rehabilitation in chronic lung disease. A randomized controlled trial. *Chest* 2001;119:1705-10.

37. Gottlieb V, Lyngsø AM, Nybo B, et al Pulmonary rehabilitation for moderate COPD (GOLD 2) - does it have an effect?. *COPD* 2011;8(5):380-6.

38. Verulava T, Jorbenadze R, Barkalaia T. Introduction of universal health program in Georgia: Problems and Perspectives. *Georgian medical news* 2017;262:116–20.

For peer review only - http://bmjopen.bmj.com/site/about/guidelines.xhtml

39. Sddharthan T, Wosu AC, Pollard SL, et al. A novel case-finding instrument for chronic obstructive pulmonary disease in low- and middle-income country settings. *Int J Chron Obstruct Pulmon Dis* 2020;15 :2769-2777

40. Kaur I, Aggarwal B, Gogtay J. Understanding perception of chronic obstructive pulmonary disease among general practitioners, physicians, and pulmonologists in India: Results from a face-to-face survey. *Perspect Clin Res* 2016;7:100-5.

41. Dennis S, Reddel HK, Middleton S, et al. Barriers and outcomes of an evidence-based approach to diagnosis and management of chronic obstructive pulmonary disease (COPD) in Australia: A qualitative study. *Family Practice* 2017;34(4):485-490 doi:10.1093/fampra/cmw103.

42. Haroon S, Jordan RE, Fitzmaurice DA, et al. Case finding for COPD in primary care: a qualitative study of the views of health professionals. *Int J Chron Obstruct Pulmon Dis* 2015:10 1711–1718.

43. Keating A, Lee A, Holland A. What prevents people with chronic obstructive pulmonary disease from attending pulmonary rehabilitation? A systematic review. *Chron Respir Dis* 2011;8(2):89-99.

44. Steiner M, McMillan V, Lowe D, et al. Pulmonary rehabilitation: steps to breathe better. Pulmonary rehabilitation: An exercise in improvement. National Chronic Obstructive Pulmonary Disease (COPD) Audit Programme: Clinical and organisational audits of pulmonary rehabilitation services in England and Wales 2017. National report. London: RCP, April 2018. https://www.nacap.org.uk/nacap/welcome.nsf/vwFiles/COPD-PR-2017-Clinical-and-organisational/$File/Pulmonary%20Rehabilitation%20-%20An%20exercise%20in%20improvement%20-%20National%20report%20(Web).pdf?openelement (accessed 06/07/2021).

45. Oates GR, Niranjan SJ, Ott C, et al. Adherence to Pulmonary Rehabilitation in COPD: A qualitative exploration of patient perspectives on barriers and facilitators. *J Cardiopulm Rehabil Prev* 2019;39(5):344-349.

46. Bjoernshave B, Korsgaard J, Nielsen CV. Does pulmonary rehabilitation work in clinical practice? A review on selection and dropout in randomized controlled trials on pulmonary rehabilitation. *Clin Epidemiol* 2010;2:73–83.

47. Steiner MC, Roberts CM, Lowe D, et al. on behalf of the National COPD Audit programme: pulmonary rehabilitation workstream 2013–18. Pulmonary Rehabilitation: Time to breathe better. November 2015. https://www.rcplondon.ac.uk/projects/outputs/pulmonary-rehabilitation-time-breathe-better (date accessed 06/06/21).

48. Polastri M, Nava S, Clini E, et al. COVID-19 and pulmonary rehabilitation: preparing for phase three *ERJ* 2020;55(6):2001822 doi: 10.1183/13993003.01822-2020.

For peer review only - http://bmjopen.bmj.com/site/about/guidelines.xhtml

49. Wang TJ, Chau B, Lui M, Physical Medicine and Rehabilitation and Pulmonary Rehabilitation for COVID-19. *Am J Phys Med Rehabil*. 2020;99(9):769-774 doi:10.1097/PHM.0000000000001505.

50. Demeco A, Marotta N, Barletta M, et al. Rehabilitation of patients post-COVID-19 infection: a literature review. *J Int Med Res* 2020;48(8) doi: 10.1177/0300060520948382.

51. Hansen H, Bieler T, Beyer N, et al. Supervised pulmonary tele-rehabilitation versus pulmonary rehabilitation in severe COPD: A randomised multicentre trial. *Thorax* 2020;75:421 doi:10.1136/thoraxjnl-2019-214246.

52. Holland AE, Malaguti C, Hoffman M, et al. Home-based or remote exercise testing in chronic respiratory disease, during the COVID-19 pandemic and beyond: A rapid review. *Chron Respir Dis*. 2020;17:1479973120952418.

For peer review only

For peer review only - http://bmjopen.bmj.com/site/about/guidelines.xhtml

Figure legend

Figure 1: CONSORT Flow diagram

For peer review only

For peer review only - http://bmjopen.bmj.com/site/about/guidelines.xhtml

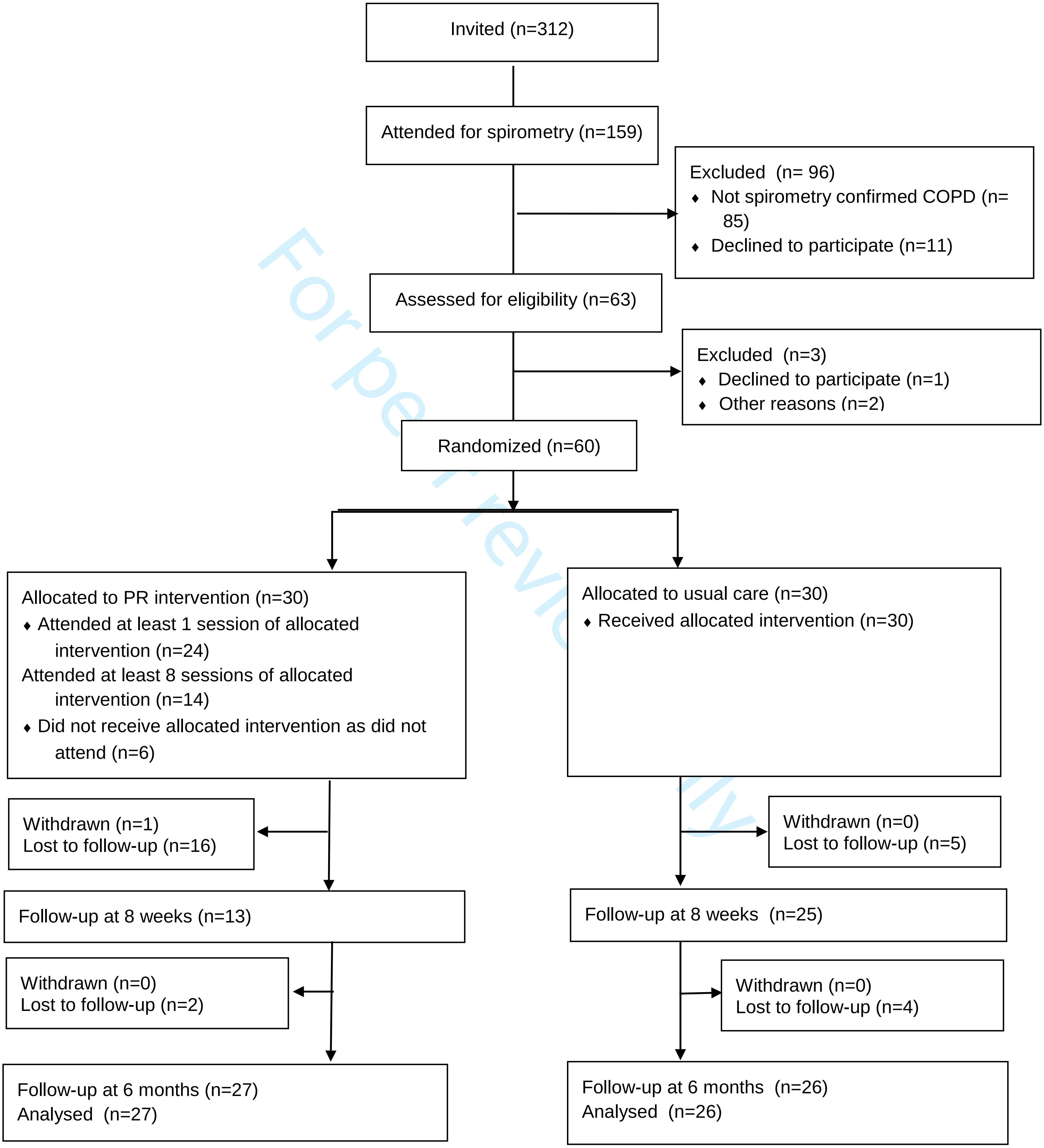

For peer review only

Supplementary tables and figures

Table S1: List of education topics delivered as part of the PR programme

COPD as a global problem
What you and your family should know about COPD
Breathing and breath physiology
Tobacco, its impact on health and smoking cessation
Benefits of physical activity
Regular exercises
Clearance of the respiratory system: keeping clean lungs
Energy conservation: additional energy
Home oxygen therapy
COPD and swallowing
Management of depression, anxiety and fears during COPD
Nutrition recommendations for COPD patients
Self-assessment and self-management
Loving relationships/Sexuality
COPD and incontinence
COPD and treatment, drugs

For peer review only

Table S2: Topics covered in training the rehabilitation specialist

Overview of pulmonary rehabilitation
information about the incremental shuttle walk test (ISWT) test and practical
Patient assessment
Difference between cardiac and pulmonary rehabilitation.
Format of a pulmonary rehab programme.
Exercise prescription.
Practical session of the exercises.
Education sessions:<br>anxiety and breathlessness,<br>airway clearance techniques (ACBT),<br>relaxation
Discharge from rehab to include:<br>Assessment,<br>Goal setting<br>Maintenance exercise.

For peer review only

Table S3: Costs of Pulmonary rehabilitation (PR) delivery

Type of cost	Units	Unit cost (GBP)	Total cost (GBP)
**Equipment costs:**			
TheraBand Resistive	5	£4.60	£23.00
Theraband Resistive	5	£5.00	£25.00
Theraband Resistive	5	£4.40	£4.00
Stepbox 2x	2	£15.00	£31.00
Weights 10kg	2	£13.00	£26.00
Weights 4kg	2	£5.30	£10.60
Medicine ball	2	£22.00	£44.00
Medicine ball	2	£14.00	£28.00
Water Dispenser	1	£96.00	£96.00
Stopwatch timer	8	£5.00	£40.00
Bottled water	3	£3.00	£9.60
Plastic cups	3	£0.40	£1.00
**Equipment total**		**£187.70**	**£338.20**
**Intervention development:**			
Translation patient video into Georgian	1		£212.00
Nutritionists' and psychologist's videos	1		£595.00
**Intervention development total**			**£807.00**
**Training:**			
Translation during PR training	1	£196.00	£196.00
Trainers - 4 days from UK	£7,200		£7,200.00
Attendees - 4 days	2	£320.00	£640.00
**Training total**			**£8,036.00**
**PR delivery**			41 sessions
Rehab specialist (£10/hr)	2 x 1.5 hours/session		
	30 mins x 7 per 16 week cycle	£10.00	£35.00
Senior respiratory specialist (£70/hr)	30 mins x 8 per 16 week cycle	£35.00	£140.00
Doctor (£40/hr)	30 mins x 1 per 16 week cycle	£20.00	£10.00
Baseline ISWT x 2	40 mins	£6.67	£200.10
Pre-rehab session	1 hr with rehab specialist	£10.00	£25.00
Exercise sessions	2 x 1.5 hours/session	£30.00	£1,230
Education	30 mins/session	£10.67	£437.47
Booklet	30	£1.80	£54.00
**Total PR delivery**			**£1,946.57**
**Total PR delivery per attendee**			**£81.11**

For peer review only

Table S4: Topic guides for qualitative interviews

Patient interview guide

<u>Feasibility trial of an adapted pulmonary rehabilitation programme in Georgia</u>

1) I'd like you to tell me about your experience of taking part in this study.

2) Can you tell be about how you found out about the study?

Prompt:

a. What were your thoughts when you were invited to participate? Why did you decide to participate?

b. What ideas did you have about how it would affect your condition?

c. Did you think you would gain anything?

d. Did you have any concerns about participating?

3) What were your expectations of the PR programme prior to the start?

Prompts:

a. Had you ever heard of it before?
b. What did you think would happen in the programme?
c. How did you feel about exercise before you started the programme?

i. What type of exercise did you feel able to do then? Why?

ii. And what did you feel that you couldn't do? Why?

d. What did you expect to get from the programme? Did you think anything would change?

4) What was your experience of the research assessment visits?

Prompt:

a. What did you think of the questionnaire content/length?

b. What did you think of the shuttle walk test?

i. Did you understand what to do?
ii. How easy was it to do?

5) Can you tell me about the pre-rehab session?

For peer review only

Prompt:

a. What role did it play with regard to your understanding of the PR program?

b. What was positive about it?

c. What was less helpful?

6) Can you tell me about your experience of the rehabilitation?

Prompt:

a. What was good about it?
b. What was not so good?

7) How many of the sessions were you able to attend?

Prompt:

a. If not all of them, why not?
(Location, transport, access, cost, season, illness, family matters, holidays)

Barriers to attendance:

b. If did attend, what difficulties did you face in attending?
c. Why did you still try to keep on going?

8) What did you think of the education sessions?

Prompt:

a. COPD disease, causes [looking for smoking], treatment, exacerbations, exercise, self-management, diet, lifestyle related information [anything else]?
b. Was there anything about the education sessions/ that you received which you felt was not relevant for your condition and situation?

9) In what ways did the program influence your everyday lifestyle?

a. Have you done any of the exercises at home? If yes, are you still continuing to do them?
b. Are you a smoker? [if not already stated]
   i. Did you learn anything about smoking and COPD? [if yes]
   ii. What did you learn?

10) Did you incur any costs in attending the rehabilitation?

a. Have you made any purchases as a result of doing the rehabilitation?

Prompt: e.g. equipment? Shoes? Clothing?

11) What changes would you make to improve PR?

Prompt:

For peer review only

a. Time, location, content.
b. Was there anything you would like to add to the sessions (physical exercises of some kind, information)?
c. Anything which you think should not have been included?

12) How widely do you think pulmonary rehab should be available? *If yes…*

Prompt

a. How do you think it should be paid for? [is this something you would pay for]
b. Where and when should the sessions be?

13) What do you think are the advantages of PR in comparison with other ways of getting exercise? And the disadvantages?

14) So what are you going to do now that the rehabilitation programme is over?

Prompts around continued exercise

a. If not already discussed: Have you thought about exercising at home?
b. Do you think you will do anything to change your physical activity level and approaches now that the PR classes are over?

Ending the interview: Is there anything else you would like to say? Any important issues not raised? Thank participant for their time.

For peer review only

Specialists' Interview topic guide - Georgia

Feasibility trial of an adapted pulmonary rehabilitation programme in Georgia

1) Before participating in this study, what were your thoughts on pulmonary rehab?
   Prompts:
   a. What did you know about PR? How detailed was the information you possessed before on PR? What impact you thought it would have on patients?
   b. Impact on health? Other?
2) What are your views about pulmonary rehab (open question)
   Prompts:
   a. What experience did you have of PR?
   b. Two consecutive questions:
      a. Can you describe the positive aspects of PR from your point of view?
      b. Can you describe negative aspects of PR from your point of view?
      c. From your point of view what kind of impact does pulmonary rehab have on health?

3) Why did you agree to support this pulmonary rehab research study?
   Prompt:
   a. What were your thoughts when you were invited to take part?

4) How did you find the experience of providing PR?
   Prompt:
   a) In your opinion, as a physiotherapist what did you get from your experience?
   b) What was good about it?
   c) What was not so good?

5) How did patients seem to receive this programme?
   a) What was good?
   b) What was not so good?

6) How would you describe the effects of the PR for the patients?
   a. What benefits did you see?
   b. What challenges did you observe?

7) Can you tell me about the practical side of delivering the programme?
   a. What worked well?
   b. What didn't work so well?
   c. And from the research point of view? (was it a difficult, interesting – the meetings, the paperwork, fitting the PR appointments in around your everyday work schedule etc?)

8) What did you enjoy about delivering the programme?

9) Are there any changes you think should be made to the programme?

Prompt:

a. Time, location, content.
b. Was there anything you would like to add to the sessions?
c. Anything you would like to take out?

Future:

10) Do you think pulmonary rehab should be widely available?

If yes…

a) Where and when should the sessions be?
b) How do you think it should be paid for?

If no….

a) Can you name the reasons why?

11) How would you feel if you were asked to run regular PR programs?

12) How interesting do you think this topic is for physiotherapists?

Prompt:

a) For what reasons?

13) Can you tell me about any conversations you had about PR with your colleague physiotherapists?

Prompt:

a) How would you describe their attitude towards PR?
b) If you have not had any conversation, why do you think this topic never came up in the discussion?

14) From your point of view how will your participation in the study affect your practice?

Ending the interview: Is there anything else you would like to say? Any important issues not raised? Thank participant for their time.

For peer review only

Table S5: Post-hoc per-protocol analysis: 8 week follow-up of intervention patients who attended 50% or more pulmonary rehabilitation sessions compared to usual care.

	Intervention n=9		Usual care n=26	
	**Mean (sd)**	**Mean change (sd) [95% CI]**	**Mean (sd)**	**Mean change (sd) [95% CI]**
SGRQ - Total	27.23 (24.11)	-31.92 (23.57) [-50.05, -13.81]	48.04 (20.75)	-0.53 (17.96) [-8.12, 7.04]
SGRQ - Impacts	17.65 (20.91)	-30.03 (22.10) [-47.02, -13.03]	37.58 (23.17)	0.26 (22.85) [-9.17, 9.69]
SGRQ - Activity	37.79 (31.52)	-37.42 (26.43) [-57.74, -17.10]	58.60 (20.32)	1.13 (17.73) [-6.18, 8.45]
SGRQ - Symptoms	36.87 (29.66)	-27.94 (33.56) [-53.74, -2.15]	60.47 (25.68)	-4.16 (21.39) (-13.19, 4.86]
MRC score	2.22 (1.09)	-1.33 (1.00)	2.96 (1.09)	-0.24 (0.59)
CAT	11.44 (6.44)	-9.11 (5.66)	18.32 (7.03)	-1.36 (5.00)
PHQ9 depression score	2.11 (1.05)	-2.44 (3.20)	5.54 (5.09)	-0.29 (3.89)
GAD anxiety score	1.66 (1.41)	-1.1 (3.51)	3.32 (3.47)	-0.28 (2.80)
Stanford Self Efficacy Score	8.16 (0.32)	2.07 (1.06)	6.9 (1.52)	0.24 (1.76)
ISWT	337.77 (106.86)	146.66 (81.70)	247.6 (113.51)	-13.2 (110.66)
Self-reported number of exacerbations in the last 6 months	3.66 (1.00)	0.66 (0.86)	3.56 (0.96)	1.32 (1.67)
Total Physical Activity Scores. MET-minutes/week	1032.96 (1393.17)	27.39 (1718.72)	1634.68 (1532.01)	-1393.05 (2838.34)

For peer review only

Figure S1: Proportion of intervention group who attended pulmonary rehabilitation sessions

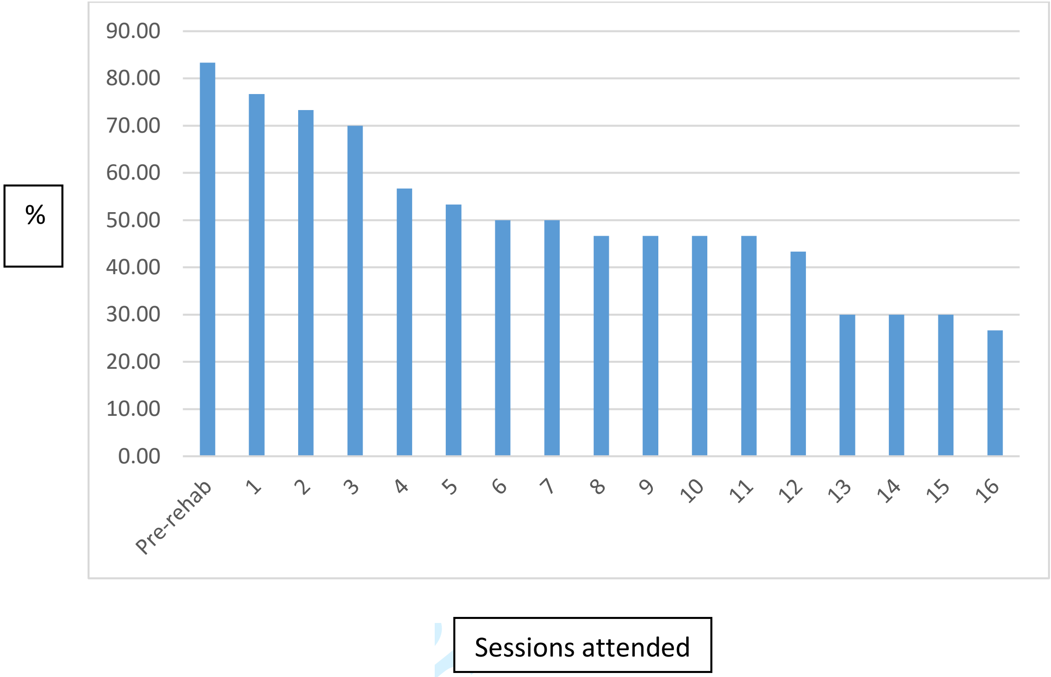

46.7% of participants attended at least 50% of planned sessions; 43% attended at least 75% of planned sessions.

For peer review only

CONSORT 2010 checklist of information to include when reporting a pilot or feasibility trial*

Section/Topic	Item No	Checklist item	Reported on page No
**Title and abstract**			
	1a	Identification as a pilot or feasibility randomised trial in the title	1
	1b	Structured summary of pilot trial design, methods, results, and conclusions (for specific guidance see CONSORT abstract extension for pilot trials)	2
**Introduction**			
Background and objectives	2a	Scientific background and explanation of rationale for future definitive trial, and reasons for randomised pilot trial	4
	2b	Specific objectives or research questions for pilot trial	4 aim – add objectives?
**Methods**			
Trial design	3a	Description of pilot trial design (such as parallel, factorial) including allocation ratio	4
	3b	Important changes to methods after pilot trial commencement (such as eligibility criteria), with reasons	n/a
Participants	4a	Eligibility criteria for participants	4-5
	4b	Settings and locations where the data were collected	4
	4c	How participants were identified and consented	5
Interventions	5	The interventions for each group with sufficient details to allow replication, including how and when they were actually administered	5-6
Outcomes	6a	Completely defined prespecified assessments or measurements to address each pilot trial objective specified in 2b, including how and when they were assessed	7
	6b	Any changes to pilot trial assessments or measurements after the pilot trial commenced, with reasons	n/a
	6c	If applicable, prespecified criteria used to judge whether, or how, to proceed with future definitive trial	-
Sample size	7a	Rationale for numbers in the pilot trial	5
	7b	When applicable, explanation of any interim analyses and stopping guidelines	n/a
Randomisation:			
Sequence generation	8a	Method used to generate the random allocation sequence	5
	8b	Type of randomisation(s); details of any restriction (such as blocking and block size)	5
Allocation concealment mechanism	9	Mechanism used to implement the random allocation sequence (such as sequentially numbered containers), describing any steps taken to conceal the sequence until interventions were assigned	5

For peer review only

Implementation	10	Who generated the random allocation sequence, who enrolled participants, and who assigned participants to interventions	Needs more detail
Blinding	11a	If done, who was blinded after assignment to interventions (for example, participants, care providers, those assessing outcomes) and how	?
	11b	If relevant, description of the similarity of interventions	n/a
Statistical methods	12	Methods used to address each pilot trial objective whether qualitative or quantitative	Table for objectives and outcomes?
**Results**			
Participant flow (a diagram is strongly recommended)	13a	For each group, the numbers of participants who were approached and/or assessed for eligibility, randomly assigned, received intended treatment, and were assessed for each objective	Fig 1
	13b	For each group, losses and exclusions after randomisation, together with reasons	Fig 1
Recruitment	14a	Dates defining the periods of recruitment and follow-up	6
	14b	Why the pilot trial ended or was stopped	n/a
Baseline data	15	A table showing baseline demographic and clinical characteristics for each group	11
Numbers analysed	16	For each objective, number of participants (denominator) included in each analysis. If relevant, these numbers should be by randomised group	15
Outcomes and estimation	17	For each objective, results including expressions of uncertainty (such as 95% confidence interval) for any estimates. If relevant, these results should be by randomised group	15
Ancillary analyses	18	Results of any other analyses performed that could be used to inform the future definitive trial	15
Harms	19	All important harms or unintended effects in each group (for specific guidance see CONSORT for harms)	18
	19a	If relevant, other important unintended consequences	n/a
**Discussion**			
Limitations	20	Pilot trial limitations, addressing sources of potential bias and remaining uncertainty about feasibility	21
Generalisability	21	Generalisability (applicability) of pilot trial methods and findings to future definitive trial and other studies	21-22
Interpretation	22	Interpretation consistent with pilot trial objectives and findings, balancing potential benefits and harms, and considering other relevant evidence	20
	22a	Implications for progression from pilot to future definitive trial, including any proposed amendments	22
**Other information**			
Registration	23	Registration number for pilot trial and name of trial registry	3
Protocol	24	Where the pilot trial protocol can be accessed, if available	3
Funding	25	Sources of funding and other support (such as supply of drugs), role of funders	24

For peer review only

	26	Ethical approval or approval by research review committee, confirmed with reference number	24

Citation: Eldridge SM, Chan CL, Campbell MJ, Bond CM, Hopewell S, Thabane L, et al. CONSORT 2010 statement: extension to randomised pilot and feasibility trials. BMJ. 2016;355.
*We strongly recommend reading this statement in conjunction with the CONSORT 2010, extension to randomised pilot and feasibility trials, Explanation and Elaboration for important clarifications on all the items. If relevant, we also recommend reading CONSORT extensions for cluster randomised trials, non-inferiority and equivalence trials, non-pharmacological treatments, herbal interventions, and pragmatic trials. Additional extensions are forthcoming: for those and for up to date references relevant to this checklist, see www.consort-statement.org.

For peer review only

BMJ Open

Feasibility of a pulmonary rehabilitation programme for patients with symptomatic chronic obstructive pulmonary disease in Georgia: a single site, randomized controlled trial from the Breathe Well Group

Journal:	*BMJ Open*
Manuscript ID	bmjopen-2021-056902.R1
Article Type:	Original research
Date Submitted by the Author:	10-May-2022

Complete List of Authors:	Maglakelidze, Mariam; Georgian Respiratory Association; Petre Shotadze Tbilisi Medical Academy Ltd<br>Kurua, Ia; Georgian Respiratory Association<br>Maglakelidze, Nino; Georgian Respiratory Association<br>Maglakelidze, Tamaz; Ivane Javakhishvili Tbilisi State University Faculty of Medicine; Tbilisi State Medical University<br>Chkhaidze, Ivane; Georgian Respiratory Association; Tbilisi State Medical University Faculty of Medicine<br>Gogvadze, Ketevan; Georgian Respiratory Association<br>Chkhaidze, Natia; M. Iashvili Children's Central Hospital<br>Beadle, Helen; University Hospitals Birmingham NHS Foundation Trust, Department of Physiotherapy<br>Redden-Rowley, Kelly; Sandwell and West Birmingham Hospitals NHS Trust, iCares Directorate<br>Adab, Peymane; University of Birmingham College of Medical and Dental Sciences, Institute of Applied Health Research<br>Adams, Rachel; University of Birmingham College of Medical and Dental Sciences, Institute of Applied Health Research<br>Chi, Chunhua; Peking University First Hospital, General Practice Department, Peking University First Hospital, Beijing, 100034, China<br>Cheng, Kar Keung; University of Birmingham College of Medical and Dental Sciences, Institute of Applied Health Research<br>Cooper, Brendan; University Hospitals Birmingham NHS Foundation Trust, Lung Function & Sleep<br>Correia-de-Sousa, Jaime; University of Minho, Life and Health Sciences Research Institute; Horizonte Family Health Unit,<br>Dickens, Andrew; University of Birmingham, Institute of Applied Health Research<br>Enocson, Alexandra; University of Birmingham, Applied Health Research<br>Farley, Amanda; University of Birmingham College of Medical and Dental Sciences, Institute of Applied Health Research<br>Gale, Nicola K.; University of Birmingham College of Arts and Law, Health Services Management Centre<br>Jowett, Sue; University of Birmingham, Health Economics Unit<br>Martins, Sonia; ABC Medical School, Family Medicine<br>Rai, Kiran; University of Birmingham College of Medical and Dental Sciences, Institute of Applied Health Research<br>Sitch, Alice; University of Birmingham College of Medical and Dental

	Sciences, Institute of Applied Health Research; University Hospitals Birmingham NHS Foundation Trust and University of Birmingham, NIHR Birmingham Biomedical Research Centre<br>Stavrikj, Katarina; Medical Faculty, Centre for Family Medicine<br>Stelmach, Rafael; University of São Paulo<br>Turner, Alice; University of Birmingham College of Medical and Dental Sciences, Institute of Applied Health Research<br>Williams, Sian; National Heart and Lung Institute<br>Jordan, Rachel; University of Birmingham College of Medical and Dental Sciences, Institute of Applied Health Research<br>Jolly, Kate; University of Birmingham College of Medical and Dental Sciences, Institute of Applied Health Research

**Primary Subject Heading**:	Respiratory medicine
Secondary Subject Heading:	Rehabilitation medicine
Keywords:	Thoracic medicine < INTERNAL MEDICINE, INTERNAL MEDICINE, GENERAL MEDICINE (see Internal Medicine), Chronic airways disease < THORACIC MEDICINE, REHABILITATION MEDICINE, Clinical trials < THERAPEUTICS

SCHOLARONE™ Manuscripts

I, the Submitting Author has the right to grant and does grant on behalf of all authors of the Work (as defined in the below author licence), an exclusive licence and/or a non-exclusive licence for contributions from authors who are: i) UK Crown employees; ii) where BMJ has agreed a CC-BY licence shall apply, and/or iii) in accordance with the terms applicable for US Federal Government officers or employees acting as part of their official duties; on a worldwide, perpetual, irrevocable, royalty-free basis to BMJ Publishing Group Ltd ("BMJ") its licensees and where the relevant Journal is co-owned by BMJ to the co-owners of the Journal, to publish the Work in this journal and any other BMJ products and to exploit all rights, as set out in our licence.

The Submitting Author accepts and understands that any supply made under these terms is made by BMJ to the Submitting Author unless you are acting as an employee on behalf of your employer or a postgraduate student of an affiliated institution which is paying any applicable article publishing charge ("APC") for Open Access articles. Where the Submitting Author wishes to make the Work available on an Open Access basis (and intends to pay the relevant APC), the terms of reuse of such Open Access shall be governed by a Creative Commons licence – details of these licences and which Creative Commons licence will apply to this Work are set out in our licence referred to above.

Other than as permitted in any relevant BMJ Author's Self Archiving Policies, I confirm this Work has not been accepted for publication elsewhere, is not being considered for publication elsewhere and does not duplicate material already published. I confirm all authors consent to publication of this Work and authorise the granting of this licence.

For peer review only

Feasibility of a pulmonary rehabilitation programme for patients with symptomatic chronic obstructive pulmonary disease in Georgia: a single site, randomized controlled trial from the Breathe Well Group

Maglakelidze Mariam[1,2], Ia Kurua[1], Nino Maglakelidze[1], Tamaz Maglakelidze[1,3*], Ivane Chkhaidze[1,4], Ketevan Gogvadze[1], Natia Chkhaidze[5], Helen Beadle[6], Kelly Redden-Rowley[7], Peymané Adab[8], Rachel Adams[8], Chunhua Chi[9], Kar Keung Cheng[8], Brendan G Cooper[10], Jaime Correia-de-Sousa[11,12], Andrew P Dickens[8], Alexandra Enocson[8], Amanda Farley[8], Nicola Gale[13], Sue Jowett[8], Sonia M Martins[14], Kiran Rai[8], Alice Sitch[8,15], Katarina Stavrikj[16], Rafael Stelmach[17], Alice Turner[8], Siân Williams[11], Rachel E Jordan[8], Kate Jolly[8]

*Corresponding author: Tamaz Maglakelidze (tmaglak@gmail.com);

[1]Georgian Respiratory Association, 2/6, Liubliana str, Tbilisi, Georgia.

[2]Petre Shotadze Tbilisi Medical Academy, 51/2 Ketevan Dedofali Ave, Tbilisi, 0144, Georgia.

[3]Ivane Javakhishvili Tbilisi State University, 1 Ilia Chavchavadze Avenue, Tbilisi, 0179, Georgia.

[4]Tbilisi State Medical University, 33 Vazha Pshavela Ave, Tbilisi, Georgia.

[5]M. Iashvili Children's Central Hospital, Lubliana str 13/6, Tbilisi, 0159, Georgia.

[6]Department of Physiotherapy, University Hospitals NHS Foundation Trust, Solihull Hospital, Solihull, B91 2JL, UK.

[7]Community Cardio-Respiratory Clinical Lead, iCares Directorate, Sandwell and West Birmingham NHS Trust, West Bromwich B71 4HJ, UK

[8]Institute of Applied Health Research, University of Birmingham, Birmingham, B15 2TT, UK

[9]Department of General Practice, Peking University First Hospital, Beijing, China

[10]Lung Function & Sleep, Queen Elizabeth Hospital, Birmingham, B15 2TH, UK

[11]International Primary Care Respiratory Group, 19 Armour Mews, Larbert, FK5 4FF, Scotland

[12] Life and Health Sciences Research Institute, School of Medicine, University of Minho. ICVS/3B's, PT Government Associate Laboratory, Braga/Guimarães, 4710-057, Portugal

[13]Health Services Management Centre, School of Social Policy, University of Birmingham, Birmingham, B15 2TT, UK

[14]Family Medicine, ABC Medical School, Sao Paolo, Brazil

[15]NIHR Birmingham Biomedical Research Centre, University Hospitals Birmingham NHS Foundation Trust and University of Birmingham, Birmingham, B15 2TT, UK

[16]Centre for Family Medicine, Medical Faculty, Skopje, Macedonia

For peer review only - http://bmjopen.bmj.com/site/about/guidelines.xhtml

[17]Faculty of Medicine, University of São Paulo, 05403-000, Brazil

Word count: 4300

For peer review only

For peer review only - http://bmjopen.bmj.com/site/about/guidelines.xhtml

ABSTRACT

Objectives: To assess the feasibility of delivering a culturally tailored pulmonary rehabilitation programme and conducting a definitive randomised controlled trial.

Design: A two-arm, randomised feasibility trial with a mixed-methods process evaluation.

Setting: Secondary care setting in Georgia, Europe

Participants: People with symptomatic spirometry confirmed COPD recruited from primary and secondary care.

Interventions: Participants were randomised in a 1:1 ratio to a control group or intervention comprising 16 twice-weekly group pulmonary rehabilitation sessions tailored to the Georgian setting.

Primary and secondary outcome measures: Feasibility of the intervention *and* RCT were assessed according to: study recruitment, consent and follow-up, intervention fidelity, adherence and acceptability, using questionnaires and measurements at baseline, programme-end and 6-months, and through qualitative interviews.

Results: The study recruited 60 participants (as planned): 54 (90%) were male, 37 (62%) had an FEV1% predicted of ≤50%. The mean MRC dyspnoea score was 3.3 (SD 0.5), and mean SGRQ 50.9 (SD 17.6). The rehabilitation specialists delivered the pulmonary rehabilitation with fidelity. Thirteen (43.0%) of participants attended at least 75% of the 16 planned sessions. Participants and rehabilitation specialists in the qualitative interviews reported that the programme was acceptable, but drop-out rates were high in participants who lived outside Tbilisi and had large distances to travel. Outcome data was collected on 63.3% at 8 weeks and 88.0% at 6 months. Mean change in SGRQ total was -24.9 [95% CI -40.3, -9.6] at programme end and -4.4 [95% CI -12.3, 3.4] at 6 months follow-up for the intervention group and -0.5 [-8.1, 7.0] and -8.1 [-16.5, 0.3] for the usual care group at programme end and 6 months respectively.

Conclusions: The tailored pulmonary rehabilitation intervention was feasible to deliver. Approaches to improve uptake and adherence warrant further research.

Trial registration: ISRCTN16184185

For peer review only - http://bmjopen.bmj.com/site/about/guidelines.xhtml

Key words: feasibility trial; pulmonary rehabilitation; COPD; middle-income country; cultural adaptation

Strengths and limitations of this study

- This is the first published pulmonary rehabilitation trial undertaken in Georgia.
- The intervention was culturally tailored for a middle income country, having been selected through a structured prioritisation process involving policy makers, clinicians and patients.
- The 63% follow-up in the intervention group at 8-weeks affects interpretation.
- A post-hoc per-protocol analysis explored the outcomes in intervention participants who attended at least 50% of the pulmonary rehabilitation sessions, showing a consistent pattern of improvements in health related quality of life
- Recruitment through primary care proved challenging due to patients with COPD diagnosis from primary care not fulfilling diagnostic criteria on spirometry.

For peer review only

INTRODUCTION

Chronic obstructive pulmonary disease (COPD) is a progressive chronic inflammatory lung disease that has a significant health and economic burden, with high health care costs arising largely from hospital admissions for exacerbations.[1-3] It is currently the third leading cause of death worldwide[2] with 251 million cases reported globally in 2016.[4] Although most of these deaths occur in low and middle income countries (LMICs),[5] most research on COPD management has been undertaken in high-income countries. The burden of chronic respiratory disease (CRD) is a considerable challenge for Georgia's healthcare system with the mortality attributable to CRD about 4% in Georgia,[6] and as elsewhere in the world, rates are increasing mainly due to air pollution (outdoor and indoor from solid fuel cooking and heating;[6 7]) and high rates of tobacco use[2] (about one third of adults are current smokers). The availability of pharmacotherapy is limited due to resource constraints, making pharmacological management difficult.[8]

In addition to the direct health and healthcare burden, COPD negatively impacts the health-related quality of life (HRQoL) of people living with the condition.[9] There is growing evidence that pulmonary rehabilitation (PR) is an effective and cost-effective therapeutic intervention to improve COPD symptoms, patients' quality of life[10] and to reduce risk of death when delivered early following hospital admission for exacerbation.[11] However, evidence of the effectiveness of PR for COPD in patients from LMICs is extremely limited, barriers to provision include infrastructure for diagnosis, essential drug availability, lack of skilled health professionals, and overall healthcare priorities, which limit management options[12] and few studies have adapted PR to the local context in LMICs.[12-14] Additionally, local adaptation (or tailoring) has been reported as an enabler of PR in LMICs.[15] There are currently no PR services offered to patients in Georgia, a middle income country. In this context, where resources are limited, yet COPD is a major burden, research is needed to evaluate whether offering PR is feasible and to assess whether the intervention would have similar effects to those observed in other settings. This study evaluated the feasibility of a future trial to assess the effectiveness of a culturally tailored PR programme in Georgia.

METHODS

Study design and participants

The study was a two-arm, non-blinded, randomised feasibility trial of an adapted PR programme, with a mixed-methods process evaluation conducted in the Chapidze Emergency Cardiology Center, Tbilisi. Participants with an MRC dyspnoea score ≥2, and a spirometry-confirmed COPD diagnosis, defined as a post-bronchodilator FEV_1/FVC ratio <0.70, were deemed eligible for the study. Exclusion

For peer review only - http://bmjopen.bmj.com/site/about/guidelines.xhtml

criteria were the presence of musculoskeletal or neurological conditions preventing exercise, unstable cardiovascular disease (e.g. unstable angina, aortic valve disease, unstable pulmonary hypertension), severe cognitive impairment, severe psychotic disturbance, active contagious infectious disease, and very poor balance.

Sample size

We aimed to recruit 60 participants. The sample size was chosen to enable estimation of feasibility outcomes with reasonable precision.[16] A follow-up rate of 80% could be estimated with a precision of 68% to 89% (binomial exact 95% confidence interval).

Recruitment and randomisation

Participants were recruited (December 2018- May 2019) from six primary care facilities and three secondary care facilities in Tbilisi; all facilities provided services to patients without geographical restriction. An information brochure about the programme was distributed among doctors in primary care facilities and doctors from the Georgian Respiratory Association working in secondary healthcare. These facilities provided contact details of patients with COPD, who were contacted via telephone. The MRC dyspnoea score[17] was completed over the telephone and those with a score of ≥2 were invited to the spirometry eligibility assessment. Before spirometry, patients were given a study participant information leaflet. Post-bronchodilator spirometry was performed according to American Thoracic Society and European Respiratory Society 2005 guidelines[18] using a Spirolab III spirometer and over-read by a member of the research team (AE or BC). Eligible participants were asked to give informed, written consent to participate in all aspects of the trial, including qualitative interviews. Randomisation to one of the two groups in a 1:1 ratio was performed using an electronic database (REDcap).[19 20]

Intervention

The intervention was adapted from PR programmes delivered in the UK, and following UK and ERS/ATS guidance.[21 22] Three focus groups took place in Tbilisi, Georgia and involved people with COPD (n=6), family practitioners and respiratory physicians (n=6), and rehabilitation specialists (n=7) who discussed potential components for the PR programme. Their views were considered alongside the APEASE criteria[23] (Affordability, Practicality, Effectiveness and cost-effectiveness, Acceptability, Side-effects/Safety, Equity). Adaptations were made to the programme, taking into consideration contextual, cultural and resource needs in Georgia. Given the high literacy rates of the population, an information booklet about COPD, covering the 16 topics included in the PR education sessions, was also produced in the local language. The main adaptations included recognition of the

For peer review only - http://bmjopen.bmj.com/site/about/guidelines.xhtml

importance of including family members, by inviting them to attend a pre-rehabilitation information session and the educational talks. The educational sessions did not include discussions of end of life or the potentially terminal nature of COPD as requested by focus group participants.

A pre-rehabilitation one-to-one assessment was booked at a time convenient for the patient. The aim of this assessment was to orientate the patient to the PR intervention and tailor their exercise programme. During this visit, patients were given a pulmonary rehabilitation information booklet, created for the trial by the research team. The distance walked in the incremental shuttle walk test (ISWT),[24] Borg rating of perceived exertion and modified Borg breathlessness scales[25] were used to prescribe the intensity of the exercise component of the PR programme. Participants had pulse-oximetry measured during the ISWT and were prescribed 65 to 85% of their maximal baseline ISWT performance (lower in deconditioned patients). Changes were not made to patients' medication.

The adapted PR programme drew on international guidance[21 22] and consisted of two main components: 1½ hours of exercise which took place in a hospital rehabilitation gym and 15-20 minutes of education on the management of COPD covering 16 topics (see Supplementary Table S1 for additional information on intervention content). The description, content and frequency of these components were determined during the intervention development phase of this project (cultural tailoring). The programme took place twice weekly for an eight week period as a rolling programme (i.e. new patients were introduced at any point).

The intervention was delivered by two rehabilitation specialists (physiotherapists), who had not previously delivered PR. They were trained by UK respiratory physiotherapists to deliver PR over two visits and received a total of four days of training. Training topics are detailed in Supplementary Table S2.

Usual care group

Participants randomised to the control group received usual care from their primary care doctor and or pulmonologist. They were offered a delayed 1.5 - 2 hour educational session delivered by pulmonologists and specialists once the final (6-months) follow-up was complete. During this session, participants received the same PR educational booklet as the intervention group.

Outcome measures

Data were collected from patients by the research team members at the end of the PR programme (eight weeks) and at six months, at a clinical assessment; participants who were unable or unwilling to attend the assessment were invited to complete the questionnaires over the telephone. Follow-

For peer review only - http://bmjopen.bmj.com/site/about/guidelines.xhtml

up was not blinded. A baseline questionnaire captured sociodemographic and health-related characteristics of the participants.

Feasibility outcomes

The main feasibility outcomes were delivery of the PR with fidelity, acceptability to participants, recruitment rate, follow-up rate at six months, adherence to the intervention, ability to carry out trial procedures, feasibility of methods to measure costs of PR, other COPD-related health care utilisation (including number of hospital admissions since enrolment) and patient-incurred costs.

Secondary outcomes

Secondary outcomes included the primary outcome of a future definitive RCT, which was the St George's Respiratory Questionnaire (SGRQ)[26] at six months (total, symptoms, activity and impact). Other secondary outcome measures at eight weeks and six months were: SGRQ at programme end, exercise capacity measured by the ISWT (metres),[24] smoking status validated by cotinine, COPD Assessment Test (CAT),[27] self-reported physical activity (IPAQ),[28] self-efficacy measured by the Stanford Self-Efficacy scale,[29] depression (PHQ-9)[30] anxiety (GAD-7),[31] and self-reported number of exacerbations (in the last six months).

To assess the feasibility of collecting outcome data for a future health economic analysis we collected the EQ-5D-5L,[32] self-reported COPD related health care utilisation and participant incurred costs from an open question in the eight week questionnaire. In order to determine whether it would be possible to estimate the cost of PR in a future trial we recorded the number of sessions attended by each participant and total number of sessions delivered.

Process evaluation

Process measures to determine the feasibility of intervention delivery and study processes included number of PR sessions attended, fidelity of delivery, and participant engagement assessed by observation of 16 sessions. Completion of PR was defined as attendance at 75% of sessions.[33]

One rehabilitation session was observed by a member of the research team and exercise sheets were analysed to make sure they were delivered with fidelity. Fidelity was defined as evidence of increasing exercise prescriptions and recorded activities over the PR course for three aerobic and three strength exercises.

We telephoned patients who had agreed to be contacted for interview, using purposive sampling to increase the mix of socio-demographic characteristics, severity of COPD and differing levels of

For peer review only - http://bmjopen.bmj.com/site/about/guidelines.xhtml

engagement with the PR programme. After completion of the programme, semi-structured interviews were conducted with nine participants (who attended between two and sixteen PR sessions) and the two rehabilitation specialists to assess their experience of it. Participant interviews were conducted by telephone, and face to face with the rehabilitation specialists. Structured topic guides (see supplementary table S3) were used and refined iteratively as themes emerged. The average interview duration was 35 minutes for the patient participants and 33 minutes for the rehabilitation specialists. Sample size was determined by thematic saturation.[34]

Data analysis

Analyses concentrated on available data only, with no attempt made to impute missing values. Analyses commenced once the last participant completed follow up. Participant demographics were tabulated to understand the population recruited and whether recruitment reflected the socio-demographic profile of people with COPD in Georgia. We report recruitment and follow-up rates, with 95% confidence intervals, overall and by study arm. Although the trial was not powered to detect a difference between intervention and wait-list control, we calculated the mean change in SGRQ at PR end and 6 months for those allocated to each group; 95% confidence intervals were provided for estimates obtained. We provided estimates of the secondary outcomes by group and display binary outcomes using counts and percentages, and continuous data are presented using means and standard deviations. Since the study
was not powered to detect treatment effects on clinical outcomes, p values and 95% CIs were not reported. Data were analysed using STATA/IC 15.1.

To explore whether the lack of effect at six months in the intervention group was due to lack of sustained effect or lack of engagement with PR by many of the intervention participants a post-hoc per protocol analysis was undertaken. Outcomes of intervention group participants who attended at least 50% of sessions[35] (n=13) were calculated.

Audio recordings of qualitative interviews were transcribed intelligent verbatim, anonymised and analysed using content analysis.[36]

For peer review only - http://bmjopen.bmj.com/site/about/guidelines.xhtml

Patient and public involvement

A research prioritisation exercise was initially conducted with patients, clinicians and policy makers; the need to find effective, affordable COPD management that suited Georgian priorities and processes was selected. A patient advisory group in the UK advised on PR design, highlighting the importance of sessions being local with minimal travel costs. The intervention adaptation had input from six patients who had COPD. The study was overseen by a Trial Steering Committee, which included a person with COPD, who was involved throughout all stages of the study, contributing to decisions by commenting on intervention process, materials, advising on recruitment approaches and dissemination of findings.

RESULTS

Feasibility of conducting a definitive RCT

Participant recruitment and follow-up

We contacted 312 patients in order to recruit 60 for the study. After meeting primary criteria for study inclusion, 159 patients underwent spirometry, of whom 74 had COPD confirmed. Sixty-three patients were assessed for eligibility and attended the baseline visit, 60 were recruited and randomised (30 intervention and 30 usual care) (figure 1). One participant withdrew from the intervention group; follow-up at six months was 88% across both groups. Recruitment from primary care was challenging with the majority (n=53, 88.3%) of participants coming from a hospital setting. Participants who were identified in primary care and assessed for eligibility were frequently excluded following spirometry.

Baseline characteristics of participants

Participant characteristics are detailed in Table 1. Overall, 90% of participants were male, the mean age was 65 years (SD 7.95); most were Georgian (n=56; 93.3%). Twenty-three (38.3%) were current smokers and all had at least one comorbidity. Only 20 (33.3%) were in paid work. Few participants (n=10, 16.7%) were not using any COPD medication. The mean (SD) MRC dyspnoea score was 3.28 (0.52) and 37 (61.7%) had an FEV1% predicted of ≤50% indicating severe COPD. The mean SGRQ was 50.87 (SD 17.59).

For peer review only - http://bmjopen.bmj.com/site/about/guidelines.xhtml

Table 1: Baseline characteristics of the patients for intervention and usual care group.

**Characteristic**	**Intervention N=30**	**Usual care N=30**	**All N=60**
Sex, n (%) Male	26 (86.7%)	28 (93.3%)	54 (90.0%)
Age in years, mean (SD)	64 (7.3)	65 (8.7)	65 (8)
Ethnicity, n (%)			
Georgian	28 (93.3)	28 (93.3)	56 (93.3%)
Other ethnic group	2 (6.7)	2 (6.7)	4 (6.7%)
Education, n (%)			
No formal qualification	0 (0.0)	2 (6.67)	2 (3.3)
High school education	7 (23.3)	6 (20.0)	13 (21.7)
Degree level or higher	15 (50.0)	14 (46.7)	29 (48.3)
Other	8 (26.7)	8 (26.7)	16 (26.7)
Living alone, n(%)	3 (10.0)	4 (13.3)	7 (11.7)
Relationship status. n (%)			
Married and living with partner	22 (73.3)	22 (73.3)	4 (73.3)
Never married	3 (10.0)	4 (13.3)	7(11.67)
Separated, but still legally married	1 (3.3)	1 (3.3)	2 (3.3)
Divorced	4 (13.3)	1 (3.3)	5 (8.3)
Widowed	0 (0.0)	2 (6.67)	2 (3.3)
Employment status, n (%)			
In paid work (full or part-time including self-employed)	7 (23.3)	13 (43.3)	20 (33.3)
Unemployed/looking for work	10 (33.3)	2 (6.7)	12 (20.0)
Retired from paid work	11 (36.7)	11 (36.7)	22 (36.7)
Looking after the family or home	10 (33.3)	6 (20.0)	16 (26.7)
Unable to work because of my chest/lung problem	5 (16.7)	1 (3.3)	6 (10.0)
Unable to work because of other long term health problem	2 (6.7)	1 (3.3)	3 (5.0)
Other	6 (20.0)	5 (16.7)	11(18.3)
Co-morbidities – ever diagnosed, n(%)			
Diabetes	5 (16.7)	2 (6.7)	7 (11.7)
High blood pressure	11 (36.7)	11 (36.7)	22 (36.7)
Coronary heart disease/Angina/Heart Attack	6 (20.0)	4 (13.3)	10 (16.7)
Heart failure	7 (23.3)	7 (23.3)	14 (23.3)
Stroke/mini-stroke	0 (0.0)	4 (13.3)	4 (6.7)
Asthma	7 (23.3)	5 (16.7)	12 (20.0)
Tuberculosis	4 (13.3)	2 (6.7)	6 (10.0)
Osteoarthritis	5 (16.7)	4 (13.3)	9 (15.0)
Osteoporosis	1 (3.3)	1 (3.3)	2 (3.3)
Depression	2 (6.7)	2 (6.7)	4 (6.7)
Other condition	5 (16.7)	5 (16.7)	10 (16.7)
Medication use			

For peer review only

For peer review only - http://bmjopen.bmj.com/site/about/guidelines.xhtml

SABA/SAMA	9 (30.0)	6 (20.0)	15 (25.0)
LABA/LAMA	10 (33.3)	15 (50.0)	25 (41.7)
ICS	2 (6.7)	2 (6.7)	4 (6.7)
ICS/LABA	15 (50.0)	17 (56.7)	32 (53.3)
Steroids	2 (6.7)	1 (3.3)	3 (5.0)
Methylxanthine	2 (3.3)	2 (3.3)	4 (6.7)
Other	9 (30.0)	9 (30.0)	18 (30.0)
None of the above	6 (20.0)	4 (13.3)	10 (16.7)
Smoking status n (%)			
Current	10 (23.3)	13 (43.3)	23 (38.3)
Ex-smoker	22 (73.4)	21 (70.0)	43 (71.7)
Never smoker	2 (6.67)	2 (6.7)	4 (6.67)
GOLD stage, n (%)			
IV (FEV_1 <30% predicted)	1 (3.3)	1 (3.3)	2 (3.3)
III (FEV_1 30-49% predicted)	4 (13.3)	4 (13.3)	8 (13.3)
II (FEV_1 50-79% predicted)	19 (63.3)	18 (60.0)	37 (61.7)
I (FEV_1 ≥80% predicted)	6 (20.0)	7 (23.3)	13 (21.7)
MRC dyspnoea Score, mean, (SD)	3.4 (0.6)	3.2 (0.4)	3.3 (0.5)
MRC score 3, n (%)	21 (70.0)	24 (80.0)	45 (75.0)
MRC score 4, n (%)	7 (23.3)	6 (20.0)	13 (21.7)
MRC score 5, n (%)	2 (6.7)	0 (0.0)	2 (3.3)
COPD Assessment Test (CAT) score, mean (SD)	20.3 (5.9)	19.9 (6.6)	20.1 (6.2)
PHQ-9 depression score, mean (SD)	6.2 (4.0)	5.3 (4.1)	5.8 (4.0)
GAD-7 anxiety score, mean (SD)	3.5 (3.2)	3.3 (2.8)	3.4 (3.0)
Stanford Self Efficacy Score, mean (SD)	5.7 (1.9)	6.7 (1.5)	6.2 (1.7)
ISWT, mean (SD)	229.7 (112.7)	249.3 (96.2)	239.5 (104.3)
Self-reported number of exacerbations in the last 6 months, mean (SD)	2.7 (0.9)	2.3 (1.1)	2.5 (1.1)
St Georges Respiratory Questionnaire (SGRQ)			
SGRQ - Impact, mean (SD)	42.0 (19.9)	37.0 (20.3)	39.5 (20.1)
SGRQ - Activity, mean (SD)	67.0 (20.2)	56.9 (24.1)	62.0 (22.6)
SGRQ - Symptoms, mean (SD)	64.2 (17.1)	65.4 (15.0)	64.8 (16.0)
SGRQ - Total, mean (SD)	53.6 (17.2)	48.1 (17.9)	50.9 (17.6)
Total Physical Activity Scores, MET-minutes/week (SD)	2092.8 (2647.2)	3027.7 (3303.9)	2560.3 (3005.3)

FEV1 – forced expiratory volume in 1 second; GAD: generalized anxiety Disorder-7; ICS: inhaled corticosteroid; LABA: long-acting beta agonist; LAMA: long-acting muscarinic antagonist; MET: metabolic equivalent of task; PHQ9: Patient Health Questionnaire-9; SABA: short-acting beta agonist; SAMA: short-acting muscarinic antagonist; SD: standard deviation.

Follow-up rates

For peer review only - http://bmjopen.bmj.com/site/about/guidelines.xhtml

Follow up rates at eight weeks were 63.3% (38); but were much lower in the intervention arm (13; 43.3%) compared to the usual care group (25; 83.3%). Participants who did not complete follow-up at eight weeks had similar characteristics to the full sample and did not differ by smoking status, SGRQ or PHQ9 score. Follow-up at 6 months was 88.3% (n=53); seven participants were administered the questionnaire by telephone, so did not undertake the ISWT, hand grip strength or cotinine measurement. One participant completed the questionnaire by telephone but attended the assessments.

Completeness of follow-up questionnaires

The level of completion of the questionnaire was high in all cases where the questionnaire was completed at the assessment and over the phone with 100% completion of questions.

Feasibility of economic evaluation in definitive RCT

The EQ-5D-5L and self-reported COPD-related health care utilisation questions had high levels of completion. Intervention participants reported costs mainly related to transportation (bus, petrol fuel, taxi). The cost of the programme came up as an important determinant for attendance in the future. Most participants noted that they would not be able to attend PR if they had to pay for it. A small number noted they would be able to pay 15-20 euros or 10% of the fee. Equipment costs, number of sessions delivered and staff costs for PR session delivery were established; the component costs are detailed in Supplementary Table S4.

Outcomes of a future RCT

At programme end (eight weeks), those with follow-up data in the intervention group (n=13) showed substantial improvements across all the outcomes including the SGRQ and ISWT, with mean changes (SD) of -24.9 (25.4) [95%CI -40.3, -9.6] and 120.8m (89.5) respectively. The usual care group (n=26) showed only minimal changes (Table 2). At the six month follow-up improvements in the intervention (n=27) were still visible, but much more modest. The usual care group (showed considerable improvements at the six month follow-up for all the outcomes except for the ISWT and exacerbations (Table 2)**.**

For peer review only - http://bmjopen.bmj.com/site/about/guidelines.xhtml

Table 2: Effect of pulmonary rehabilitation at 8 weeks and 6 months for patients in the intervention and usual care group

	8 week follow-up				6 month Follow-up			
	Intervention		Usual care		Intervention		Usual care	
	**Mean (SD) N=13**	**Mean change (SD) [95% CI]**	**Mean (SD) N=25**	**Mean change (SD) [95% CI]**	**Mean (SD) N=27**	**Mean change from baseline (SD) [95% CI]**	**Mean (SD) N=26**	**Mean change from baseline (SD) [95% CI]**
SGRQ - Total[a]	32.3 (27.4)	-24.9 (25.4) [-40.3, -9.6]	48.0 (20.8)	-0.5 (18.0) [-8.1, 7.0]	48.1 (23.5)	-4.4 (19.8) [-12.3, 3.4]	40.8 (20.0)	-8.1 (20.8) [-16.5, 0.3]
SGRQ - Impacts	23.3 (25.6)	-22.7 (22.6) [-36.4, -9.1]	37.6 (23.2)	0.3 (22.9) [-9.2, 9.7]	38.4 (23.6)	-2.4 (21.0) [-10.7, 5.9]	30.1 (20.0)	-7.5 (25.5) [-17.7, 2.9]
SGRQ - Activity	42.5 (32.9)	-30.5 (29.4) [-48.3, -12.8]	58.6 (20.3)	1.1 (17.7) [-6.2, 8.5]	58.5 (29.8)	-7.8 (26.5) [-18.3, 2.7]	49.2 (24.8)	-10.5 (23.3) [-19.9, -1.1]
SGRQ - Symptoms	41.0 (31.6)	-21.6 (37.3) [-44.1, 1.0]	60.5 (25.7)	-4.2 (21.4) [-13.2, 4.9]	58.1 (23.4)	-4.5 (24.1) [-14.1, 5.0]	57.7 (24.8)	-6.1 (22.4) [-15.1, 3.1]
MRC dyspnoea Score	2.5 (1.2)	-0.9 (1.3)	3.0 (0.6)	-0.2 (0.6)	2.9 (1.0)	-0.4 (1.0)	2.8 (0.9)	-0.3 (0.8)
COPD Assessment Test (CAT) score[b]	13.8 (8.5)	-6.6 (7.4)	18.3 (7.0)	-1.4 (5.0)	17.9 (8.5)	-1.7 (7.3)	17.7 (7.6)	-2.7 (6.5)
PHQ9 depression score[c]	2.8 (1.9)	-2.8 (2.9)	5.5 (5.1)	-0.3 (3.9)	4.74 (4.2)	-1.2 (3.8)	3.8 (3.0)	-1.9 (3.2)
GAD anxiety score[c]	1.9 (1.6)	-0.9 (3.0)	3.3 (3.5)	-0.3 (2.8)	2.51 (3.3)	-0.7 (2.8)	2.0 (2.1)	-1.3 (2.6)
Stanford Self Efficacy Score	7.8 (1.2)	1.9 (1.5)	6.9 (1.5)	0.3 (1.8)	7.0 (1.5)	1.2 (2.1)	7.3 (1.2)	0.7 (1.5)
ISWT[d]	316.2 (134.3)	120.8 (89.5)	247.6 (113.5)	-13.2 (110.7)	217.6 (89.6)	-7.1 (92.5)	216 (99.4)	-29.2 (74.1)
Number of exacerbations in the last 6 months	3.5 (1.1)	0.7 (1.3)	3.6 (1.0)	1.3 (1.7)	3.2 (1.3)	0.5 (2.5)	3.2 (1.3)	0.92 (1.7)

For peer review only - http://bmjopen.bmj.com/site/about/guidelines.xhtml

Total Physical Activity Scores. MET-minutes/week (SD)	783.2 (1509.9)	-1309.6 (2539.0)	1634.7 (1532.0)	-1393.1 (2838.3)	943.6 (1163.5)	-1653.3 (4650.6)	1857.0 (2853.4)	-2386.7 (5537.9)

[a]A reduction in SGRQ is a positive patient outcome, minimal clinically important difference (MCID) 4 points; [b]MCID: -2 points; [c]MCID 20% reduction; [d]MCID 35-36m

Outcomes in relation to smoking are reported in Table 3. At the 8-week follow-up none of the participants had quit smoking, but two of the intervention and three of the usual care group had made quit attempts. By the 6-month follow-up, one of the intervention group and two of the usual care group had quit smoking and an additional four of each group had made quit attempts.

Table 3: Self-reported tobacco use at 8 weeks and 6 months

	**8 week FU**		**6 month FU**	
**Tobacco use**	**Intervention N=13 n (%)**	**Usual care N=25 n (%)**	**Intervention N=27 n (%)**	**Usual care N=26 n (%)**
Current smoker at study start	3 (23.1%)	10 (40.0%)	9 (33.3%)	9 (34.6%)
Current smoker at follow-up	3 (23.1%)	8 (32.0%)	10 (37.0%)	7 (26.9%)
Smokers at baseline who tried to quit since enrolling in the research study	2 (66.7%)	3 (30.0%)	4 (44.4%)	4 (44.4%)
Smokers at baseline who quit since enrolling in the trial (cotinine validated)	0 (0%)	1 (10.0%)	1 (11.1%)	2 (22.2%)

Attendance and adherence

Eight participants attended all 16 sessions, 13 (43.3%; or 54.2% of those who attended at least one session) attended at least 75% and six did not attend any sessions (supplementary figure 1). Participants gave several reasons for not attending PR sessions particularly highlighting “cold weather, family issues and not being in town”.

For peer review only - http://bmjopen.bmj.com/site/about/guidelines.xhtml

Per-protocol analysis

At the six month assessment only 13 intervention participants who had completed 8 (50%) or more PR sessions were followed up. Table 4 reports the per-protocol analysis at 6 months; the 8-week results are in supplementary table S5.

The SGRQ would be the primary outcome of a future RCT and at 6-months large, sustained improvements were seen across all the domains for the intervention group. A consistent pattern of sustained improvements were seen across all the outcomes, apart from the ISWT which showed a modest improvement at 6-months.

For peer review only

For peer review only - http://bmjopen.bmj.com/site/about/guidelines.xhtml

Table 4: Post-hoc per-protocol analysis: 6 month follow-up of intervention patients who attended 50% or more pulmonary rehabilitation sessions compared to usual care

	Intervention n=13		Usual care n=26	
	**Mean (SD)**	**Mean change (SD) [95% CI]**	**Mean (SD)**	**Mean change (SD) [95% CI]**
SGRQ - Total[a]	44.9 (24.3)	-16.6 (14.5) [-25.3, -7.8]	40.8 (20.0)	-8.1 (20.8) [-16.5, 0.3]
SGRQ - Impacts	36.6 (22.7)	-14.1 (16.8) [-24.3, -4.0]	30.1 (20.0)	-7.5 (25.5) [-17.7, 2.9]
SGRQ - Activity	53.3 (33.7)	-22.9 (21.3) [-35.8, -10.1]	49.2 (24.8)	-10.5 (23.3) [-19.9, -1.1]
SGRQ - Symptoms	54.7 (24.8)	-12.6 (20.1) [-24.7, -0.4]	57.7 (24.8)	-6.0 (22.4) [15.1, 3.1]
MRC dyspnoea score	2.6 (1.0)	-1.0 (0.8)	2.8 (0.9)	-0.3 (0.8)
CAT[b]	16.2 (7.1)	-5.5 (4.8)	17.7 (7.6)	-2.7 (6.5)
PHQ-9 depression score[c]	4.8 (5.0)	-0.9 (3.4)	3.8 (3.0)	-1.9 (3.2)
GAD anxiety score[c]	2.8 (3.6)	-0.8 (3.2)	2.0 (2.1)	-1.3 (2.6)
Stanford Self Efficacy Score	7.3 (1.7)	1.6 (2.0)	7.3 (1.2)	0.7 (1.5)
ISWT[d]	217.5 (85.7)	17.5 (72.6)	216 (99.4)	-29.2 (74.1)
Self-reported number of exacerbations in the last 6 months	2.9 (1.5)	0.1 (1.5)	3.2 (1.27)	0.9 (1.7)
Total Physical Activity Scores. MET-minutes/week	1078.5 (1498.5)	72.92 (1816.8)	1857.0 (2853.4)	-1170.7 (2753.3)

[a]MCID 4 points; [b]MCID: -2 points; [c]MCID 20% reduction; [d]MCID 35-36m

For peer review only - http://bmjopen.bmj.com/site/about/guidelines.xhtml

For peer review only

Intervention fidelity

Observation of one rehabilitation session and assessment of the rehabilitation specialists' records identified high levels of fidelity with the intervention delivery. In all participants there was an increase in numbers of repetitions/intensity of at least some of the exercises over their participation period.

"…their exercise intensity is increasing, number of sets is increasing," (Rehab specialist)

Intervention acceptability

Most patients had a positive experience with the exercise, specialists, educational sessions and the booklet. Education sessions and guidance by the rehabilitation specialists were highly valued.

Most patients felt satisfied with the programme; some mentioned they felt it did not influence their behaviour much, whilst some emphasised that they felt more "joyful." Transport, hot weather during summer, health, money and work-related reasons were mentioned as barriers to continuing with the PR programme. Overall, the exercises were well received but suggestions to include more breathing exercises were made.

"[my physical activity] has changed, I walk more freely. When I need to lift heavy things, it is not difficult for me. It worked very well on me, and psychologically I am very well disposed" (P2)

Barriers: "weather and my health" (P4)

"I missed last two sessions … Transportation was a challenge." (P5)

The rehabilitation specialists reported that many participants improved and that attending the rehabilitation in a group increased their motivation.

"Those who completed 16 exercise sessions have asked us to print out exercise sheets. Some of the patients who had completed the exercises asked us to continue their exercises here, since the atmosphere here was different and group exercise was increasing their motivation." (Rehab specialist)

"Many patients did not know how to manage breathing, how to go outside confidently, did not know how to manage sexual relations, nutrition, what would be beneficial and what was not necessary. COPD patients usually are thin, because of their disease. Due to physical

For peer review only - http://bmjopen.bmj.com/site/about/guidelines.xhtml

activity, one patient gained 7kg and another one 5kg, which made them very happy and they say result of the exercises." (Rehab specialist)

However, for some participants motivation was an issue as well as barriers of travel and time.

"but patients were missing sessions nevertheless. Those who had money, did not have time, and some did not work and transportation was a problem." (Rehab specialist)

Adverse events

There were no adverse events reported throughout the study.

DISCUSSION

This study aimed to determine the feasibility of delivering a culturally tailored PR programme in Georgia. Our results indicate that it was feasible to train rehabilitation specialists to deliver the PR with fidelity. There were challenges in recruiting participants from primary care, as many general practitioners do not have access to spirometry within the primary care setting. As a consequence, many patients assessed from primary care did not have an objective measure to support their diagnosis and on spirometry did not fulfil the criteria for COPD. Adherence to the programme was sub-optimal with only 50% of intervention group participants attending at least 50% of the planned sessions. The programme was acceptable to the participants who attended, but clearly the fact that 20% of intervention participants did not attend any sessions suggests that there were considerable barriers to attendance. The rehabilitation specialists reported high drop-out rates in participants who lived outside Tbilisi and had long journey times (<1 hour). Participants who attended demonstrated clinically significant improvements in outcomes, exceeding the minimally important clinical differences for the SGRQ, CAT, PHQ-9; GAD-7 and ISWT.[37-40] In a post-hoc per-protocol analysis of intervention group participants who attended at least 50% of planned sessions, the outcomes were well sustained to six months.

The Cochrane review of PR for COPD[10] reported significant improvements in all the domains of the Chronic Respiratory Questionnaire and for the SGRQ, with the total score improving more than the minimally clinically important difference of four points, which was mirrored in our study. Of the 65 RCTs included the majority were from high income settings and many of those from LMICs had small sample sizes.

For peer review only - http://bmjopen.bmj.com/site/about/guidelines.xhtml

We undertook some cultural tailoring of the PR before implementation, but did not find a need to make major changes to the programme given the delivery context in a secondary care setting. However, the discussion of end of life care was deemed unacceptable by participants from all three focus groups during the cultural adaptation, so was not included. Other researchers[41] have made significant adaptations to PR for patients who have recovered from tuberculosis, in Uganda, reporting high levels of engagement and good outcomes,[41] and in Greece,[33] and evaluated implementation in non-randomised studies. Several ongoing studies of PR in LMICs are registered on clinical trials registers.

Strengths and limitations

This study has a number of strengths. Whilst there have been many trials of PR, there are no published findings of trials undertaken in the Georgian setting. The topic was selected following a structured prioritisation process involving policy makers, clinicians and patients in Tbilisi, Georgia.[42] We undertook some cultural tailoring prior to finalising the final form of PR to be delivered to increase acceptability. The training was delivered by experienced trainers from the UK.

The study also has some limitations. A key issue was the low follow-up rate at end of programme in the intervention group resulting in an overall follow-up rate of 63% at eight weeks; similar to results reported in other studies.[43 44] The differential follow-up at 8-weeks makes the findings difficult to interpret. The fall-off in effects between eight weeks and six months in the intervention group might have been the result of more adherent participants attending the 8-week follow-up. To address this issue our post-hoc per protocol analysis explored the effects of the intervention in those participants most adherent to the PR programme and identified sustained improvements in health outcomes in this group. We faced challenges in recruiting primary care patients with COPD, largely due to the lack of availability of spirometry for diagnosis.[8] This could be overcome by alternatives to spirometry such as peak flow and questionnaires to diagnose COPD.[45] Studies from other settings have also reported structural and health care practitioner factors affecting the quality of care delivered for COPD.[46-48]

In our study only 43% would categorized as having completed the programme, i.e. attended at least 75% of the planned sessions. Adherence to PR is variable with reported non-completion rates from 42% across a range of high income settings[49] to 38% in the UK in 2017.[50] Previous PR research has reported similar barriers to our study, including transportation difficulties, financial barriers, lack of support, illness and lack of motivation.[51] The literature reports large drop-out rates between referral and pre-assessment and up to 15% of participants not continuing to PR following the pre-

For peer review only - http://bmjopen.bmj.com/site/about/guidelines.xhtml

assessment.[50 52] We randomised participants prior to the pre-rehabilitation session and 20% of our intervention participants did not commence the PR programme; other researchers have reported high levels of non-attendance with up to 50% of people referred to PR not attending a single session.[53]

Our study findings may not apply to patients with COPD managed in primary care in Georgia, as 89% of our study participants were recruited from secondary care and therefore had more severe disease. If PR is implemented in Georgia, referrals will either need to be from secondary care, or spirometry or other diagnostic methods[45] included in the referral pathway from primary care, or inclusion criteria for PR widened to include those without confirmed COPD.

Our population may not be representative of all those with COPD in Georgia as a high proportion of participants were from secondary care, had considerable symptoms (all MRC dyspnoea score 3 or more), yet 16.7% were not taking medication for their lung condition. Our qualitative sample of respondents was relatively small, but the researchers felt that thematic saturation was achieved; the sample may not have been generalizable to all those who took part.

Recommendations for future research

This study highlighted deficiencies in COPD diagnosis in primary care in Georgia and future research could explore ways to strengthen management of CRDs outside the secondary care setting.

A large trial or implementation research in the Georgian context would establish optimal delivery and effectiveness of PR for COPD. Additionally, the effectiveness of PR in Georgia for patients with other respiratory diseases and post-COVID lung damage might be undertaken.[54-56] The decision about progression to a full trial or an implementation study should draw on the feasibility outcomes of findings. Given the very minor adaptations made to the PR programme we would argue that future research should focus on best methods of implementation rather than effectiveness.

For future intervention delivery, the issue of accessibility needs to be considered, with rehabilitation provision near to where people live. The PR programme was delivered in a secondary care setting in Georgia and consideration should be given to delivery in community settings, or even home-based programmes. These could draw on the remote[57] and home-based programmes delivered in response to the COVID-19 pandemic.[58] Widespread roll-out would require training of the rehabilitation specialist workforce in Georgia. Another significant barrier is cost, as PR is not currently included within the free health care provision in Georgia. However, since this research was prioritized by

For peer review only - http://bmjopen.bmj.com/site/about/guidelines.xhtml

policy makers in Georgia there is a clear recognition that it may be valuable, offering a possible route to change this in the future.

Conclusions

These findings demonstrate the feasibility of PR delivery in Georgia and showed promising outcomes for those participants who adhered to the intervention. However, investment to enable primary care to diagnose and refer people with COPD is needed. Challenges with retention suggest that future PR programmes need to be delivered close to where people live and possibly provide flexibility in methods of delivery.

Acknowledgements:

We gratefully acknowledge:

The International Primary Care Respiratory Group for introducing us to the primary care networks involved in this study and for its continued facilitation of clinical engagement.

The Trial Steering Group Committee and the International Scientific Advisory Committee: Prof Niels Chavennes, Leiden University (Chair); Dr Semira Manaseki-Holland, University of Birmingham; Dr N. Zhvania (Professor in cardiology, Georgia); Revaz Vachnadze (patient representative) Georgia; Prof. Debbie Jarvis, Imperial College London (Chair); Dr Nega Milevsla, Centre for Regional Policy Research and Cooperation "Studiorum"; Skopje; Gordna Kunovska, Skopje.

We especially want to thank: the patients included in the study, the Chapidize Emergency Cardiology Center for providing the rehabilitation facilities, Kelly Redden-Rowley and Helen Beadle for delivering the PR training, the Georgian rehabilitation specialists Shota Bzarashvili and Teona Kvitsadze for delivering the PR - without all of you this project would not have been possible.

Finally, we would like to acknowledge Radmila Ristovska (1955-2020), also involved in the initiation of this study.

Contributors: KJ, REJ, PA co-led the study design, with contributions and advice from all other authors. TM was the local PI and oversaw all activities in Georgia. The cultural adaptation was overseen by NM, KJ, KR. The team from Georgia (MM, IK, NM, TM, IC, KG, NC) contributed to study design, day to day trial management, recruitment, intervention and follow up overseen by KJ, RA, KR, AT, REJ, PA. AE oversaw the quality of lung function testing. KR-R, HB delivered the PR training to

For peer review only - http://bmjopen.bmj.com/site/about/guidelines.xhtml

the Georgian rehabilitation specialists. NM, MM collected the qualitative data overseen by KR, RA, NG. AS, SJ designed the analysis plan and economic data collection respectively. IK conducted the statistical analysis, supported by AS, AD, KJ. KJ, MM, NM, IK wrote the manuscript with input from all other authors. As part of the Breathe Well Global Health Research Group, CC, KKC, BGC, JCS, AF, SMM, KS, RS, ~~SS~~ SW contributed to the initial topic identification, protocol development and ongoing oversight of this study. All authors contributed to and approved the final version.

Funding: This research was funded by the National Institute for Health Research (NIHR) (NIHR global group on global COPD in primary care, University of Birmingham, (project reference: 16/137/95) using UK aid from the UK Government to support global health research. KJ is part-funded by the NIHR Applied Research Collaboration West Midlands (ARCWM). The views expressed in this publication are those of the author(s) and not necessarily those of the NIHR, ARCWM or the UK Department of Health and Social Care.

Competing interests: None declared.

Patient consent for publication: Not required.

Ethical approval: The study was approved by Acad. G.Chapidze Emergency Cardiology Center, Medical Ethical Committee (session protocol №2-2018) and by the University of Birmingham ERN_18-0856.

Data availability statement: All data are available on request from the corresponding author subject to a data sharing agreement.

Figure legend

Figure 1: CONSORT Flow diagram

REFERENCES

1. May SM, Li JT. Burden of chronic obstructive pulmonary disease: healthcare costs and beyond. Allergy Asthma Proc; 2015. OceanSide Publications.

For peer review only - http://bmjopen.bmj.com/site/about/guidelines.xhtml

2. World Health Organization. Chronic obstructive pulmonary disease (COPD) [https://www.who.int/news-room/fact-sheets/detail/chronic-obstructive-pulmonary-disease-(copd) accessed 22/11/20.

3. Centers for Disease Control and Prevention. COPD Costs 2019 [https://www.cdc.gov/copd/infographics/copd-costs.html accessed 22/11/20.

4. Gómez FP, Rodriguez-Roisin R. Global Initiative for Chronic Obstructive Lung Disease (GOLD) guidelines for chronic obstructive pulmonary disease. *Curr Opin Pulm Med* 2002;8(2):81-86.

5. GBD Chronic Respiratory Disease Collaborators. Prevalence and attributable health burden of chronic respiratory diseases, 1990–2017: a systematic analysis for the Global Burden of Disease Study 2017. *Lancet Respir Med* 2020;8(6):585-96.

6. Gamkrelidze A MN, Sturua L, Demetrashvili N, Kakutia N. Non-communicable Diseases Risk-factors STEPS survey, Georgia 2016 [https://www.who.int/ncds/surveillance/steps/STEPS_Georgia_2016_ENG_summary_2016_final.pdf accessed 01/06/2021.

7. American Cancer Society. The Tobacco Atlas, Georgia Country Facts [https://tobaccoatlas.org/wp-content/uploads/pdf/georgia-country-facts.pdf accessed 22/11/20.

8. Verulava T, Jorbenadze R, Barkalaia T. Introduction of universal health program in Georgia: Problems and Perspectives. *Georgian Med News* 2017;262(1):116-20.

9. Wacker ME, Jörres RA, Karch A, et al. Assessing health-related quality of life in COPD: comparing generic and disease-specific instruments with focus on comorbidities. *BMC Pulm Med* 2016;16(1):70. doi: 10.1186/s12890-016-0238-9.

10. McCarthy B, Casey D, Devane D, et al. Pulmonary rehabilitation for chronic obstructive pulmonary disease. *Cochrane Database Syst Rev* 2015;(2): CD003793. doi: 10.1002/14651858.CD003793.pub3.

11. Ryrsø CK, Godtfredsen NS, Kofod LM, et al. Lower mortality after early supervised pulmonary rehabilitation following COPD-exacerbations: a systematic review and meta-analysis. *BMC Pulm Med* 2018;18(1):154. doi: 10.1186/s12890-018-0718-1.

12. Salvi S, Kumar GA, Dhaliwal R, et al. The burden of chronic respiratory diseases and their heterogeneity across the states of India: the Global Burden of Disease Study 1990–2016. *Lancet Glob Health* 2018;6(12):e1363-e74. doi: 10.1016/S2214-109X(18)30409-1.

13. Habib G, Rabinovich R, Divgi K, et al. Systematic review of clinical effectiveness, components, and delivery of pulmonary rehabilitation in low-resource settings. *NPJ Prim Care Respir Med* 2020;30(1):52. doi: 10.1038/s41533-020-00210-y.

14. Aït-Khaled N, Enarson D, Bousquet J. Chronic respiratory diseases in developing countries: the burden and strategies for prevention and management. *Bull World Health Organ* 2001;79:971-79.

15. Bickton FM, Shannon H. Barriers and enablers to pulmonary rehabilitation in low-and middle-income countries: a qualitative study of healthcare professionals. *Int J Chron Obstruct Pulmon Dis* 2022;17:141-53.

For peer review only - http://bmjopen.bmj.com/site/about/guidelines.xhtml

16. Lancaster GA, Dodd S, Williamson PR. Design and analysis of pilot studies: recommendations for good practice. *J Eval Clin Pract* 2004;10(2):307-12.

17. Gough J, Fletcher C. Discussion on the diagnosis of pulmonary emphysema. *Proc R Soc Med* 1952;45:576-86.

18. Miller MR, Hankinson J, Brusasco V, et al. Standardisation of spirometry. *Eur Respir J* 2005;26(2):319-38.

19. Harris PA, Taylor R, Minor BL, et al. The REDCap consortium: Building an international community of software platform partners. *J Biomed Inform* 2019;95:103208.

20. Harris PA, Taylor R, Thielke R, et al. Research electronic data capture (REDCap)—a metadata-driven methodology and workflow process for providing translational research informatics support. *J Biomed Inform* 2009;42(2):377-81.

21. Bolton CE, Bevan-Smith EF, Blakey JD, et al. British Thoracic Society guideline on pulmonary rehabilitation in adults: accredited by NICE. *Thorax* 2013;68(Suppl 2):ii1-ii30.

22. Rochester CL, Vogiatzis I, Holland AE, et al. An official American Thoracic Society/European Respiratory Society policy statement: enhancing implementation, use, and delivery of pulmonary rehabilitation. *Am J Respir Crit Care Med* 2015;192(11):1373-86.

23. Michie S, Atkins L, West R. The APEASE criteria for designing and evaluating interventions. In: Michie S, Atkins L, West R, eds. The Behaviour Change Wheel: A Guide to Designing Interventions. London: Silverback Publishing 2014.

24. Singh SJ, Jones P, Evans R, et al. Minimum clinically important improvement for the incremental shuttle walking test. *Thorax* 2008;63(9):775-77.

25. Muza SR, Silverman MT, Gilmore GC, et al. Comparison of scales used to quantitate the sense of effort to breathe in patients with chronic obstructive pulmonary disease. *Am Rev Respir Dis* 1990;141(4 Pt 1):909-13.

26. Jones PW, Quirk FH, Baveystock CM, et al. A self-complete measure of health status for chronic airflow limitation: The St. George's Respiratory Questionnaire. *Am Rev Respir Dis* 1992;145(6):1321-27.

27. Jones P, Harding G, Berry P, et al. Development and first validation of the COPD Assessment Test. *Eur Respir J* 2009;34(3):648-54.

28. Craig CL, Marshall AL, Sjöström M, et al. International physical activity questionnaire: 12-country reliability and validity. *Med Sci Sports Exerc* 2003;35(8):1381-95.

29. Lorig KR, Sobel DS, Ritter PL, et al. Effect of a self-management program on patients with chronic disease. *Eff Clin Pract* 2001;4(6):256-62.

30. Kroenke K, Spitzer RL, Williams JB. The PHQ-9: validity of a brief depression severity measure. *J Gen Intern Med* 2001;16(9):606-13.

For peer review only

For peer review only - http://bmjopen.bmj.com/site/about/guidelines.xhtml

31. Spitzer RL, Kroenke K, Williams JB, et al. A brief measure for assessing generalized anxiety disorder: the GAD-7. *Arch Intern Med* 2006;166(10):1092-97.

32. Herdman M, Gudex C, Lloyd A, et al. Development and preliminary testing of the new five-level version of EQ-5D (EQ-5D-5L). *Qual Life Res* 2011;20(10):1727-36.

33. Anastasaki M, Trigoni M, Pantouvaki A, et al. Establishing a pulmonary rehabilitation programme in primary care in Greece: a FRESH AIR implementation study. *Chron Respir Dis* 2019;16:1479973119882939.

34. Glaser B, Strauss A. Grounded Theory: The discovery of grounded theory. New York: de Gruyter 1967.

35. Johnston K, Young M, Grimmer K, et al. Frequency of referral to and attendance at a pulmonary rehabilitation programme amongst patients admitted to a tertiary hospital with chronic obstructive pulmonary disease. *Respirology* 2013;18(7):1089-94.

36. Hsieh H-F, Shannon SE. Three approaches to qualitative content analysis. *Qual Health Res* 2005;15(9):1277-88.

37. Jones PW. St. George's respiratory questionnaire: MCID. *COPD: Journal of Chronic Obstructive Pulmonary Disease* 2005;2(1):75-79.

38. Kounali D, Button KS, Lewis G, et al. How much change is enough? Evidence from a longitudinal study on depression in UK primary care. *Psychol Med* 2020:1-8.

39. Evans RA, Singh SJ. Minimum important difference of the incremental shuttle walk test distance in patients with COPD. *Thorax* 2019;74(10):994-95.

40. Kon SS, Canavan JL, Jones SE, et al. Minimum clinically important difference for the COPD Assessment Test: a prospective analysis. *Lancet Respir Med* 2014;2(3):195-203.

41. Jones R, Kirenga BJ, Katagira W, et al. A pre–post intervention study of pulmonary rehabilitation for adults with post-tuberculosis lung disease in Uganda. *Int J Chron Obstruct Pulmon Dis* 2017;12:3533-3539.

42. Dickens A, Adab P, Cheng K, et al. Using a rapid prioritisation process to identify health research priorities in LMICs: Eur Respiratory Soc, 2018.

43. Finnerty JP, Keeping I, Bullough I, et al. The effectiveness of outpatient pulmonary rehabilitation in chronic lung disease: a randomized controlled trial. *Chest* 2001;119(6):1705-10.

44. Gottlieb V, Lyngsø AM, Nybo B, et al. Pulmonary rehabilitation for moderate COPD (GOLD 2)–does it have an effect? *COPD* 2011;8(5):380-86.

45. Siddharthan T, Wosu AC, Pollard SL, et al. A novel case-finding instrument for chronic obstructive pulmonary disease in low-and middle-income country settings. *Int J Chron Obstruct Pulmon Dis* 2020;15:2769-2777.

For peer review only - http://bmjopen.bmj.com/site/about/guidelines.xhtml

46. Kaur I, Aggarwal B, Gogtay J. Understanding perception of chronic obstructive pulmonary disease among general practitioners, physicians, and pulmonologists in India: Results from a face-to-face survey. *Perspect Clin Res* 2016;7(2):100-105.

47. Dennis S, Reddel HK, Middleton S, et al. Barriers and outcomes of an evidence-based approach to diagnosis and management of chronic obstructive pulmonary disease (COPD) in Australia: a qualitative study. *Fam Pract* 2017;34(4):485-90.

48. Haroon S, Jordan RE, Fitzmaurice DA, et al. Case finding for COPD in primary care: a qualitative study of the views of health professionals. *Int J Chron Obstruct Pulmon Dis* 2015;10:1711-1718.

49. Keating A, Lee A, Holland AE. What prevents people with chronic obstructive pulmonary disease from attending pulmonary rehabilitation? A systematic review. *Chron Respir Dis* 2011;8(2):89-99.

50. Steiner M, Holzhauer-Barrie J, Lowe D, et al. Pulmonary rehabilitation: steps to breathe better. Pulmonary rehabilitation: An exercise in improvement. National chronic obstructive pulmonary disease (COPD) audit programme: clinical audit of pulmonary rehabilitation services in England and Wales 2017. National COPD audit programme. *London: Royal College of Physicians* 2018. https://www.nacap.org.uk/nacap/welcome.nsf/vwFiles/COPD-PR-2017-Clinical-and-organisational/$File/Pulmonary%20Rehabilitation%20-%20An%20exercise%20in%20improvement%20-%20National%20report%20(Web).pdf?openelement accessed 06/07/2021

51. Oates GR, Niranjan SJ, Ott C, et al. Adherence to pulmonary rehabilitation in COPD: a qualitative exploration of patient perspectives on barriers and facilitators. *J Cardiopulm Rehabil Prev* 2019;39(5):344.

52. Steiner M, Holzhauer-Barrie J, Lowe D, et al. Pulmonary rehabilitation: time to breathe better. National Chronic Obstructive Pulmonary Disease (COPD) audit programme: resources and organisation of pulmonary rehabilitation services in England and Wales 2013-18 workstream. National organisational audit report. *London: Royal College of Physicians* 2015. https://www.rcplondon.ac.uk/projects/outputs/pulmonary-rehabilitation-time-breathe-better accessed 06/06/21

53. Bjoernshave B, Korsgaard J, Nielsen CV. Does pulmonary rehabilitation work in clinical practice? A review on selection and dropout in randomized controlled trials on pulmonary rehabilitation. *Clin Epidemiol* 2010;2:73.

54. Polastri M, Nava S, Clini E, et al. COVID-19 and pulmonary rehabilitation: preparing for phase three: *Eur Respir J* 2020;55(6):2001822. doi: 10.1183/13993003.01822-2020

55. Wang TJ, Chau B, Lui M, et al. Physical medicine and rehabilitation and pulmonary rehabilitation for COVID-19. *Am J Phys Med Rehabil* 2020;99(9):769-774.

56. Demeco A, Marotta N, Barletta M, et al. Rehabilitation of patients post-COVID-19 infection: a literature review. *J Int Med Res* 2020;48(8):0300060520948382. doi: 10.1177/0300060520948382

57. Hansen H, Bieler T, Beyer N, et al. Supervised pulmonary tele-rehabilitation versus pulmonary rehabilitation in severe COPD: a randomised multicentre trial. *Thorax* 2020;75(5):413-21.

For peer review only - http://bmjopen.bmj.com/site/about/guidelines.xhtml

58. Holland AE, Malaguti C, Hoffman M, et al. Home-based or remote exercise testing in chronic respiratory disease, during the COVID-19 pandemic and beyond: a rapid review. *Chron Respir Dis* 2020;17:1479973120952418.

For peer review only

For peer review only - http://bmjopen.bmj.com/site/about/guidelines.xhtml

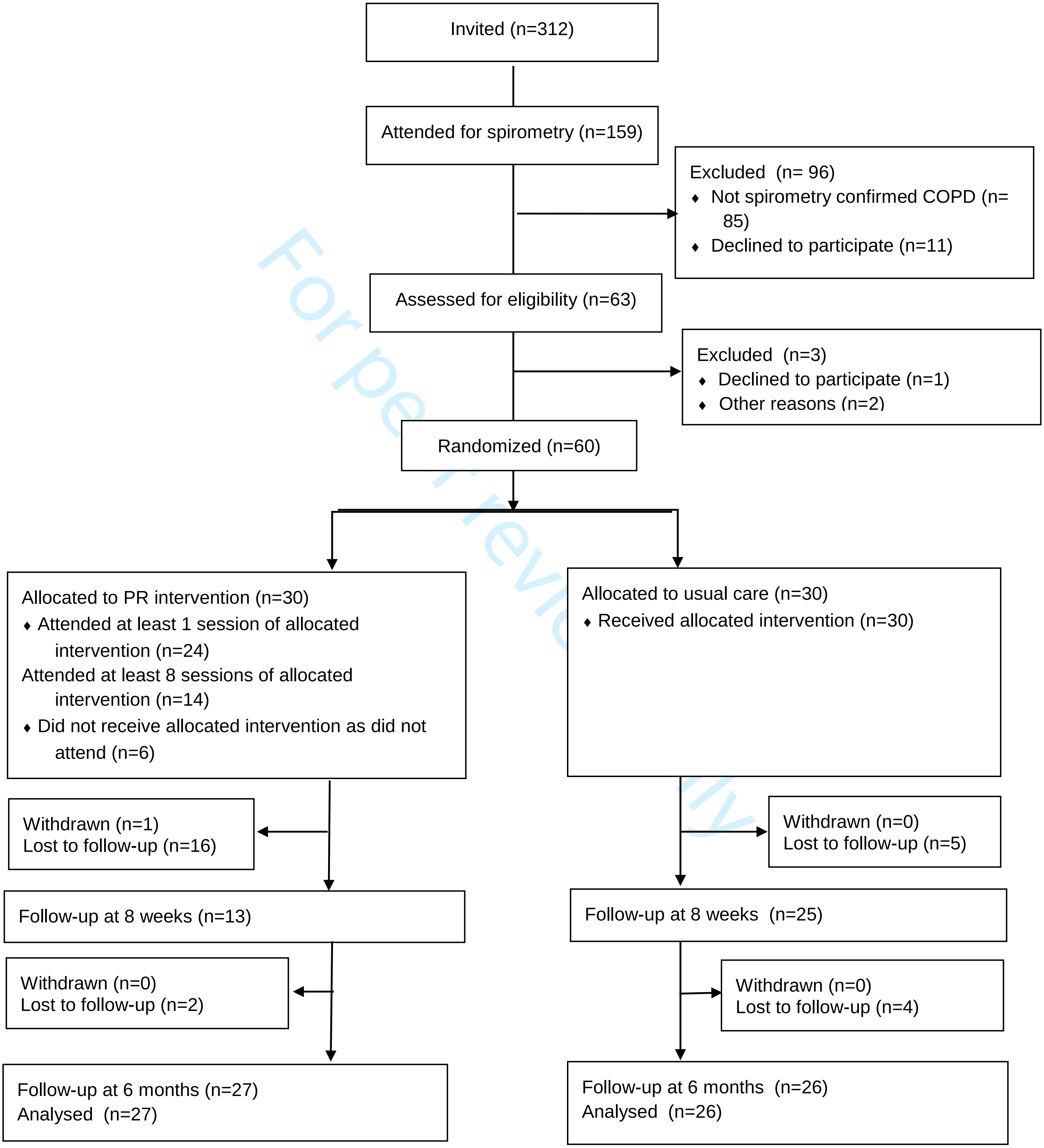

For peer review only

Supplementary tables and figures

Table S1: List of education topics delivered as part of the PR programme

COPD as a global problem
What you and your family should know about COPD
Breathing and breath physiology
Tobacco, its impact on health and smoking cessation
Benefits of physical activity
Regular exercises
Clearance of the respiratory system: keeping clean lungs
Energy conservation: additional energy
Home oxygen therapy
COPD and swallowing
Management of depression, anxiety and fears during COPD
Nutrition recommendations for COPD patients
Self-assessment and self-management
Loving relationships/Sexuality
COPD and incontinence
COPD and treatment, drugs

Components of exercise rehabilitation

Centre-based supervised exercise sessions twice weekly for 8-weeks

Advice to perform an unsupervised exercise session each week

Exercise tailored to the individual, using the modified Borg score to inform exercise prescription

Endurance and strength training

Training of upper and lower limbs

For peer review only

Table S2: Topics covered in training the rehabilitation specialist

Overview of pulmonary rehabilitation
information about the incremental shuttle walk test (ISWT) test and practical
Patient assessment
Difference between cardiac and pulmonary rehabilitation.
Format of a pulmonary rehab programme.
Exercise prescription.
Practical session of the exercises.
Education sessions:<br>anxiety and breathlessness,<br>airway clearance techniques (ACBT),<br>relaxation
Discharge from rehab to include:<br>Assessment,<br>Goal setting<br>Maintenance exercise.

For peer review only

Table S3: Topic guides for qualitative interviews

Patient interview guide

Feasibility trial of an adapted pulmonary rehabilitation programme in Georgia

1) I'd like you to tell me about your experience of taking part in this study.

2) Can you tell be about how you found out about the study?

Prompt:

a. What were your thoughts when you were invited to participate? Why did you decide to participate?

b. What ideas did you have about how it would affect your condition?

c. Did you think you would gain anything?

d. Did you have any concerns about participating?

3) What were your expectations of the PR programme prior to the start?

Prompts:

a. Had you ever heard of it before?
b. What did you think would happen in the programme?
c. How did you feel about exercise before you started the programme?

i. What type of exercise did you feel able to do then? Why?

ii. And what did you feel that you couldn't do? Why?

d. What did you expect to get from the programme? Did you think anything would change?

4) What was your experience of the research assessment visits?

Prompt:

a. What did you think of the questionnaire content/length?

b. What did you think of the shuttle walk test?

i. Did you understand what to do?
ii. How easy was it to do?

5) Can you tell me about the pre-rehab session?

For peer review only

Prompt:

a. What role did it play with regard to your understanding of the PR program?

b. What was positive about it?

c. What was less helpful?

6) Can you tell me about your experience of the rehabilitation?

Prompt:

a. What was good about it?
b. What was not so good?

7) How many of the sessions were you able to attend?

Prompt:

a. If not all of them, why not?
(Location, transport, access, cost, season, illness, family matters, holidays)

Barriers to attendance:

b. If did attend, what difficulties did you face in attending?
c. Why did you still try to keep on going?

8) What did you think of the education sessions?

Prompt:

a. COPD disease, causes [looking for smoking], treatment, exacerbations, exercise, self-management, diet, lifestyle related information [anything else]?
b. Was there anything about the education sessions/ that you received which you felt was not relevant for your condition and situation?

9) In what ways did the program influence your everyday lifestyle?

a. Have you done any of the exercises at home? If yes, are you still continuing to do them?
b. Are you a smoker? [if not already stated]
   i. Did you learn anything about smoking and COPD? [if yes]
   ii. What did you learn?

10) Did you incur any costs in attending the rehabilitation?

a. Have you made any purchases as a result of doing the rehabilitation?

Prompt: e.g. equipment? Shoes? Clothing?

11) What changes would you make to improve PR?

Prompt:

For peer review only

a. Time, location, content.
b. Was there anything you would like to add to the sessions (physical exercises of some kind, information)?
c. Anything which you think should not have been included?

12) How widely do you think pulmonary rehab should be available? *If yes…*

Prompt

a. How do you think it should be paid for? [is this something you would pay for]
b. Where and when should the sessions be?

13) What do you think are the advantages of PR in comparison with other ways of getting exercise? And the disadvantages?

14) So what are you going to do now that the rehabilitation programme is over?

Prompts around continued exercise

a. If not already discussed: Have you thought about exercising at home?
b. Do you think you will do anything to change your physical activity level and approaches now that the PR classes are over?

Ending the interview: Is there anything else you would like to say? Any important issues not raised? Thank participant for their time.

For peer review only

Specialists' Interview topic guide - Georgia

Feasibility trial of an adapted pulmonary rehabilitation programme in Georgia

1) Before participating in this study, what were your thoughts on pulmonary rehab?
   Prompts:
   a. What did you know about PR? How detailed was the information you possessed before on PR? What impact you thought it would have on patients?
   b. Impact on health? Other?
2) What are your views about pulmonary rehab (open question)
   Prompts:
   a. What experience did you have of PR?
   b. Two consecutive questions:
      a. Can you describe the positive aspects of PR from your point of view?
      b. Can you describe negative aspects of PR from your point of view?
      c. From your point of view what kind of impact does pulmonary rehab have on health?
3) Why did you agree to support this pulmonary rehab research study?
   Prompt:
   a. What were your thoughts when you were invited to take part?
4) How did you find the experience of providing PR?
   Prompt:
   a) In your opinion, as a physiotherapist what did you get from your experience?
   b) What was good about it?
   c) What was not so good?
5) How did patients seem to receive this programme?
   a) What was good?
   b) What was not so good?
6) How would you describe the effects of the PR for the patients?
   a. What benefits did you see?
   b. What challenges did you observe?
7) Can you tell me about the practical side of delivering the programme?
   a. What worked well?
   b. What didn't work so well?
   c. And from the research point of view? (was it a difficult, interesting – the meetings, the paperwork, fitting the PR appointments in around your everyday work schedule etc?)
8) What did you enjoy about delivering the programme?

For peer review only

9) Are there any changes you think should be made to the programme?

Prompt:

a. Time, location, content.
b. Was there anything you would like to add to the sessions?
c. Anything you would like to take out?

Future:

10) Do you think pulmonary rehab should be widely available?

If yes…

a) Where and when should the sessions be?
b) How do you think it should be paid for?

If no….

a) Can you name the reasons why?

11) How would you feel if you were asked to run regular PR programs?

12) How interesting do you think this topic is for physiotherapists?

Prompt:

a) For what reasons?

13) Can you tell me about any conversations you had about PR with your colleague physiotherapists?

Prompt:

a) How would you describe their attitude towards PR?
b) If you have not had any conversation, why do you think this topic never came up in the discussion?

14) From your point of view how will your participation in the study affect your practice?

Ending the interview: Is there anything else you would like to say? Any important issues not raised? Thank participant for their time.

For peer review only

Table S4: Costs of Pulmonary rehabilitation (PR) delivery

Type of cost	Units	Unit cost (GBP)	Total cost (GBP)
**Equipment costs:**			
TheraBand Resistive	5	£4.60	£23.00
Theraband Resistive	5	£5.00	£25.00
Theraband Resistive	5	£4.40	£4.00
Stepbox 2x	2	£15.00	£31.00
Weights 10kg	2	£13.00	£26.00
Weights 4kg	2	£5.30	£10.60
Medicine ball	2	£22.00	£44.00
Medicine ball	2	£14.00	£28.00
Water Dispenser	1	£96.00	£96.00
Stopwatch timer	8	£5.00	£40.00
Bottled water	3	£3.00	£9.60
Plastic cups	3	£0.40	£1.00
**Equipment total**		**£187.70**	**£338.20**
**Intervention development:**			
Translation patient video into Georgian	1		£212.00
Nutritionists' and psychologist's videos	1		£595.00
**Intervention development total**			**£807.00**
**Training:**			
Translation during PR training	1	£196.00	£196.00
Trainers - 4 days from UK	£7,200		£7,200.00
Attendees - 4 days	2	£320.00	£640.00
**Training total**			**£8,036.00**
**PR delivery**			41 sessions
Rehab specialist (£10/hr)	2 x 1.5 hours/session		
	30 mins x 7 per 16 week cycle	£10.00	£35.00
Senior respiratory specialist (£70/hr)	30 mins x 8 per 16 week cycle	£35.00	£140.00
Doctor (£40/hr)	30 mins x 1 per 16 week cycle	£20.00	£10.00
Baseline ISWT x 2	40 mins	£6.67	£200.10
Pre-rehab session	1 hr with rehab specialist	£10.00	£25.00
Exercise sessions	2 x 1.5 hours/session	£30.00	£1,230
Education	30 mins/session	£10.67	£437.47
Booklet	30	£1.80	£54.00
**Total PR delivery**			**£1,946.57**
**Total PR delivery per attendee**			**£81.11**

For peer review only

Table S5: Post-hoc per-protocol analysis: 8 week follow-up of intervention patients who attended 50% or more pulmonary rehabilitation sessions compared to usual care.

	Intervention n=9		Usual care n=26	
	**Mean (sd)**	**Mean change (sd) [95% CI]**	**Mean (sd)**	**Mean change (sd) [95% CI]**
SGRQ – Total[a]	27.23 (24.11)	-31.92 (23.57) [-50.05, -13.81]	48.04 (20.75)	-0.53 (17.96) [-8.12, 7.04]
SGRQ - Impacts	17.65 (20.91)	-30.03 (22.10) [-47.02, -13.03]	37.58 (23.17)	0.26 (22.85) [-9.17, 9.69]
SGRQ - Activity	37.79 (31.52)	-37.42 (26.43) [-57.74, -17.10]	58.60 (20.32)	1.13 (17.73) [-6.18, 8.45]
SGRQ - Symptoms	36.87 (29.66)	-27.94 (33.56) [-53.74, -2.15]	60.47 (25.68)	-4.16 (21.39) (-13.19, 4.86]
MRC dyspnoea score	2.22 (1.09)	-1.33 (1.00)	2.96 (1.09)	-0.24 (0.59)
CAT[b]	11.44 (6.44)	-9.11 (5.66)	18.32 (7.03)	-1.36 (5.00)
PHQ9 depression score[c]	2.11 (1.05)	-2.44 (3.20)	5.54 (5.09)	-0.29 (3.89)
GAD anxiety score[c]	1.66 (1.41)	-1.1 (3.51)	3.32 (3.47)	-0.28 (2.80)
Stanford Self Efficacy Score	8.16 (0.32)	2.07 (1.06)	6.9 (1.52)	0.24 (1.76)
ISWT[d]	337.77 (106.86)	146.66 (81.70)	247.6 (113.51)	-13.2 (110.66)
Self-reported number of exacerbations in the last 6 months	3.66 (1.00)	0.66 (0.86)	3.56 (0.96)	1.32 (1.67)
Total Physical Activity Scores. MET-minutes/week	1032.96 (1393.17)	27.39 (1718.72)	1634.68 (1532.01)	-1393.05 (2838.34)

[a]MCID 4 points; [b]CAT: -2 points; [c]MCID 20% reduction; [d]MCID 35-36m

For peer review only

Figure S1: Proportion of intervention group who attended pulmonary rehabilitation sessions

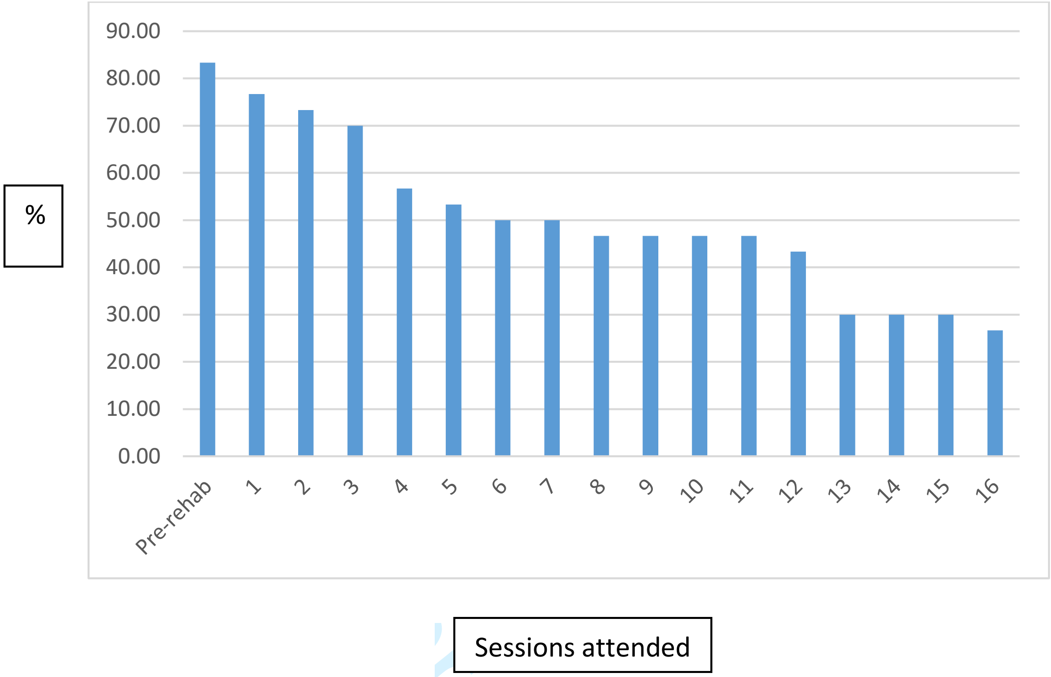

46.7% of participants attended at least 50% of planned sessions; 43% attended at least 75% of planned sessions.

CONSORT 2010 checklist of information to include when reporting a pilot or feasibility trial*

Section/Topic	Item No	Checklist item	Reported on page No
**Title and abstract**			
	1a	Identification as a pilot or feasibility randomised trial in the title	1
	1b	Structured summary of pilot trial design, methods, results, and conclusions (for specific guidance see CONSORT abstract extension for pilot trials)	2
**Introduction**			
Background and objectives	2a	Scientific background and explanation of rationale for future definitive trial, and reasons for randomised pilot trial	4
	2b	Specific objectives or research questions for pilot trial	4 aim – add objectives?
**Methods**			
Trial design	3a	Description of pilot trial design (such as parallel, factorial) including allocation ratio	4
	3b	Important changes to methods after pilot trial commencement (such as eligibility criteria), with reasons	n/a
Participants	4a	Eligibility criteria for participants	4-5
	4b	Settings and locations where the data were collected	4
	4c	How participants were identified and consented	5
Interventions	5	The interventions for each group with sufficient details to allow replication, including how and when they were actually administered	5-6
Outcomes	6a	Completely defined prespecified assessments or measurements to address each pilot trial objective specified in 2b, including how and when they were assessed	7
	6b	Any changes to pilot trial assessments or measurements after the pilot trial commenced, with reasons	n/a
	6c	If applicable, prespecified criteria used to judge whether, or how, to proceed with future definitive trial	-
Sample size	7a	Rationale for numbers in the pilot trial	5
	7b	When applicable, explanation of any interim analyses and stopping guidelines	n/a
Randomisation:			
Sequence generation	8a	Method used to generate the random allocation sequence	5
	8b	Type of randomisation(s); details of any restriction (such as blocking and block size)	5
Allocation concealment mechanism	9	Mechanism used to implement the random allocation sequence (such as sequentially numbered containers), describing any steps taken to conceal the sequence until interventions were assigned	5

For peer review only

Implementation	10	Who generated the random allocation sequence, who enrolled participants, and who assigned participants to interventions	Needs more detail
Blinding	11a	If done, who was blinded after assignment to interventions (for example, participants, care providers, those assessing outcomes) and how	?
	11b	If relevant, description of the similarity of interventions	n/a
Statistical methods	12	Methods used to address each pilot trial objective whether qualitative or quantitative	Table for objectives and outcomes?
**Results**			
Participant flow (a diagram is strongly recommended)	13a	For each group, the numbers of participants who were approached and/or assessed for eligibility, randomly assigned, received intended treatment, and were assessed for each objective	Fig 1
	13b	For each group, losses and exclusions after randomisation, together with reasons	Fig 1
Recruitment	14a	Dates defining the periods of recruitment and follow-up	6
	14b	Why the pilot trial ended or was stopped	n/a
Baseline data	15	A table showing baseline demographic and clinical characteristics for each group	11
Numbers analysed	16	For each objective, number of participants (denominator) included in each analysis. If relevant, these numbers should be by randomised group	15
Outcomes and estimation	17	For each objective, results including expressions of uncertainty (such as 95% confidence interval) for any estimates. If relevant, these results should be by randomised group	15
Ancillary analyses	18	Results of any other analyses performed that could be used to inform the future definitive trial	15
Harms	19	All important harms or unintended effects in each group (for specific guidance see CONSORT for harms)	18
	19a	If relevant, other important unintended consequences	n/a
**Discussion**			
Limitations	20	Pilot trial limitations, addressing sources of potential bias and remaining uncertainty about feasibility	21
Generalisability	21	Generalisability (applicability) of pilot trial methods and findings to future definitive trial and other studies	21-22
Interpretation	22	Interpretation consistent with pilot trial objectives and findings, balancing potential benefits and harms, and considering other relevant evidence	20
	22a	Implications for progression from pilot to future definitive trial, including any proposed amendments	22
**Other information**			
Registration	23	Registration number for pilot trial and name of trial registry	3
Protocol	24	Where the pilot trial protocol can be accessed, if available	3
Funding	25	Sources of funding and other support (such as supply of drugs), role of funders	24

For peer review only

	26	Ethical approval or approval by research review committee, confirmed with reference number	24

Citation: Eldridge SM, Chan CL, Campbell MJ, Bond CM, Hopewell S, Thabane L, et al. CONSORT 2010 statement: extension to randomised pilot and feasibility trials. BMJ. 2016;355.

*We strongly recommend reading this statement in conjunction with the CONSORT 2010, extension to randomised pilot and feasibility trials, Explanation and Elaboration for important clarifications on all the items. If relevant, we also recommend reading CONSORT extensions for cluster randomised trials, non-inferiority and equivalence trials, non-pharmacological treatments, herbal interventions, and pragmatic trials. Additional extensions are forthcoming: for those and for up to date references relevant to this checklist, see www.consort-statement.org.

For peer review only
